# Glioblastoma tumor microtubes and brain fatty acid-binding protein: Path to directional infiltration

**DOI:** 10.1093/neuonc/noaf200

**Published:** 2025-08-28

**Authors:** Won-Shik Choi, Pureunsol Jeon, Seth Peyton, Mansi Garg, John Maringa Githaka, Rong-Zong Liu, Darryl D Glubrecht, Amirali B Bukhari, Daniel McGinn, Lubna Yasmin, Caitlin Mak, Xia Xu, Matthew P Larocque, Xuejun Sun, Frank K H van Landeghem, Karolyn Au, Ing Swie Goping, Roseline Godbout

**Affiliations:** Department of Oncology, Cross Cancer Institute, University of Alberta, Edmonton, Alberta, Canada; Department of Oncology, Cross Cancer Institute, University of Alberta, Edmonton, Alberta, Canada; Department of Oncology, Cross Cancer Institute, University of Alberta, Edmonton, Alberta, Canada; Department of Oncology, Cross Cancer Institute, University of Alberta, Edmonton, Alberta, Canada; Department of Biochemistry, Faculty of Medicine and Dentistry, University of Alberta, Edmonton, Alberta, Canada; Department of Oncology, Cross Cancer Institute, University of Alberta, Edmonton, Alberta, Canada; Department of Oncology, Cross Cancer Institute, University of Alberta, Edmonton, Alberta, Canada; Department of Oncology, Cross Cancer Institute, University of Alberta, Edmonton, Alberta, Canada; Department of Oncology, Cross Cancer Institute, University of Alberta, Edmonton, Alberta, Canada; Department of Oncology, Cross Cancer Institute, University of Alberta, Edmonton, Alberta, Canada; Department of Oncology, Cross Cancer Institute, University of Alberta, Edmonton, Alberta, Canada; Department of Oncology, Cross Cancer Institute, University of Alberta, Edmonton, Alberta, Canada; Department of Oncology, Cross Cancer Institute, University of Alberta, Edmonton, Alberta, Canada; Department of Oncology, Cross Cancer Institute, University of Alberta, Edmonton, Alberta, Canada; Department of Laboratory Medicine & Pathology, University of Alberta, Edmonton, Alberta, Canada; Department of Surgery, University of Alberta, Edmonton, Alberta, Canada; Department of Biochemistry, Faculty of Medicine and Dentistry, University of Alberta, Edmonton, Alberta, Canada; Department of Oncology, Cross Cancer Institute, University of Alberta, Edmonton, Alberta, Canada

**Keywords:** fatty acid-binding protein 7, GAP43, glioblastoma, protein kinase C, tumor microtubes

## Abstract

**Background:**

Glioblastoma (GBM) is a deadly brain cancer with a dismal prognosis. There is evidence that infiltration and therapy resistance in GBM are driven by tumor microtubes (TMs), ultra-long membrane-enclosed protrusions that serve as intercellular communication channels. The aims of this study were to investigate the role of TMs and identify the molecular drivers involved in TM formation.

**Methods:**

We used patient-derived GBM neurosphere cultures that produce TMs to investigate TM dynamics, the proteins and pathways involved in TM formation, and the effect of targeting brain fatty acid-binding protein (FABP7) on mouse survival using an orthotopic model of GBM.

**Results:**

The radial glial cell marker, FABP7, is highly expressed in TMs. Like GAP43, FABP7 is critically important for the formation of TMs in GBM neurosphere cultures. We show that GBM cells use TMs as a fiber network for rapid and directional migration. Our results indicate that GAP43 phosphorylation is required for TM formation, with GAP43 phosphorylation facilitated by FABP7 expression. We also show that depletion or inhibition of protein kinase C (PKC), the kinase responsible for GAP43 phosphorylation, decreases TM formation. Targeting FABP7 in an orthotopic mouse model of TM-forming GBM cells increases survival but does not sensitize tumors to radiation.

**Conclusions:**

We found that the FABP7-PKC-pGAP43 axis is key to GBM TM formation, with TMs serving as networks for efficient long-distance cell migration. Our results indicate that TM formation can be mitigated by FABP7 inhibition with the potential of improving clinical outcomes in GBM patients.

## Graphical Abstract

**Graphical Abstract:**
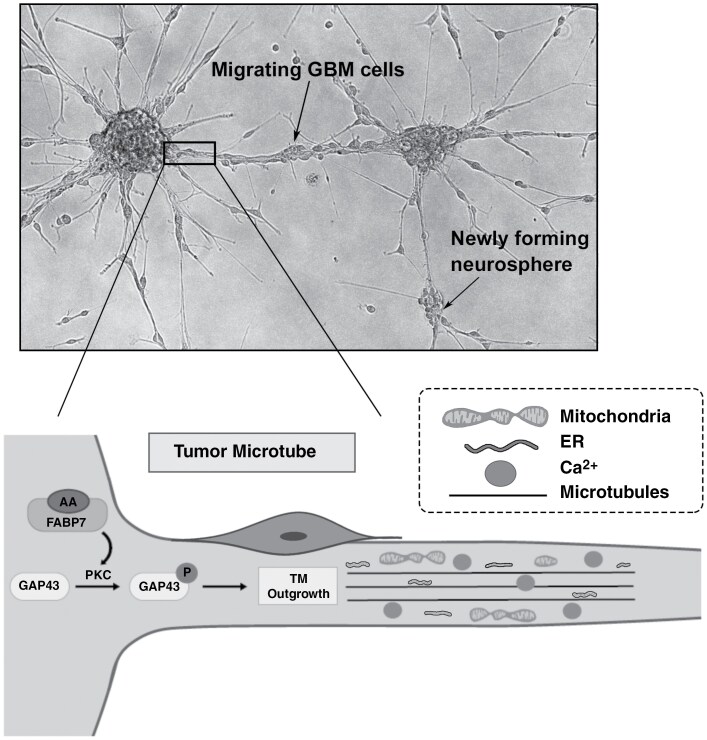

**Key Points**

- Tumor microtubes serve as migratory routes for glioblastoma cells.
- PKC inhibition or depletion prevents GAP43 phosphorylation and inhibits tumor microtube formation.
- FABP7 inhibition in an orthotopic mouse model of glioblastoma increases survival.

**Importance of the Study**

Glioblastoma (GBM) cells produce ultra-long membrane-enclosed protrusions called tumor microtubes (TMs) that contribute to therapy resistance and infiltration. Using cultures of GBM patient-derived neurospheres, we demonstrate that TMs provide a scaffold for directional and rapid movement of GBM cells. Brain fatty acid-binding protein (FABP7), a neural stem cell marker normally expressed in radial glia cells, is re-induced in GBM tumors and is abundantly expressed in TMs. FABP7 inhibition prevents TM formation, reduces cell migration, and increases sensitivity to temozolomide. Importantly, treatment of mice orthotopically injected with TM-forming GBM cells with an FABP7 inhibitor improves survival. We present a model whereby FABP7 functions through the upregulation of GAP43 phosphorylation via Protein Kinase C activation. Our findings highlight FABP7 as a promising therapeutic target for the inhibition of TM formation, which may be key to improving clinical outcomes in GBM patients.

Glioblastomas (GBMs) are highly aggressive brain cancers with a dismal prognosis.^1^ Conventional treatment using surgery, radiation therapy, and chemotherapy (usually temozolomide (TMZ)) invariably fails.^2^ These tumors are highly infiltrative and invade the surrounding brain parenchyma.^3^ The recent discovery of GBM tumor microtubes (TMs), ultra-long membrane-enclosed protrusions, has made a significant impact on our understanding of GBM biology.^4^ TMs form gap junction-coupled multicellular networks and have been shown to facilitate intercellular long-range communications through intracellular Ca^2+^ waves.^4^ TMs have been associated with resistance to both radiotherapy and chemotherapy in orthotopic mouse models of GBM.^4,5^ In addition to their contribution to therapy resistance, TMs promote GBM cell invasion by employing neuronal cell mechanisms for migration.^6^ Identifying the main players in the formation of TMs may thus be key to inhibiting GBM cell invasion and therapy resistance.

Growth-associated protein 43 (GAP43), a neuronal protein that regulates neurite formation and outgrowth, as well as neuronal plasticity,^7^ is required for TM formation in GBM, although its mechanism of action is poorly understood.^4,5^ GAP43 can be phosphorylated by protein kinase C (PKC), with phosphorylation facilitating neurite outgrowth of neuronal cells.^8,9^ Arachidonic acid (AA), an omega-6 polyunsaturated fatty acid enriched in brain, facilitates phosphorylation of GAP43 by activating PKC.^8,9^ While the regulation of GAP43 in GBM TM formation remains unclear, understanding how GAP43 is regulated may reveal new therapeutic avenues for GBM.

FABP7 is a brain fatty acid-binding protein that preferentially binds polyunsaturated fatty acids such as AA.^10^ FABP7 is highly expressed in radial glial cells during brain development.^11,12^ Radial glial cells are neural stem cells that form the fiber network along which neurons migrate to reach their final destination in the mature brain. High FABP7 levels are associated with a worse prognosis in GBM patients.^13,14^ FABP7 expression has also been linked to increased GBM cell migration and infiltration, especially in the context of an AA-rich environment.^15,16^ Inhibition of FABP7 reduces GBM neurosphere formation in vitro and inhibits tumor infiltration in an orthotopic model of GBM.^16,17^ Our results indicate that FABP7 is abundantly expressed in TM-like processes found in GBM patient tissue, mouse orthotopic models of GBM, as well as patient-derived GBM neurosphere cultures. Inhibition or depletion of FABP7 in GBM neurosphere cultures reduces TM formation and GAP43 phosphorylation. Whereas TMZ treatment induces TM formation of GBM neurosphere cultures, FABP7 inhibition attenuates TM formation in TMZ-treated GBM cultures. An orthotopic mouse model of TM-forming GBM shows significantly improved survival upon FABP7 inhibition.

## Methods

### Cell Cultures, Infections, and Reagents

GBM primary tumor tissues were collected from consented GBM patients according to the Health Research Ethics Board of Alberta Cancer Committee approval (HREBA.CC-17-0579). Tumor tissues were paraffin-embedded and enzymatically digested to generate either adherent cultures or neurosphere cultures as previously described (A4 and ED series).^18^ GBM neurosphere cells were cultured in DMEM/F12 medium supplemented with 0.5X B-27 (Life Technologies, CA), 20 ng/mL epidermal growth factor, 10 ng/mL fibroblast growth factor and 2 µg/mL heparin. Their differentiated adherent counterparts were cultured in DMEM supplemented with 10% fetal calf serum. FABP7 inhibitor (SBFI-26) was purchased from AOBIOUS Inc. (Gloucester, MA) and MedChemExpress (Monmouth Junction, NJ). Temozolomide, enzastaurin, rezasurin, and PMA were purchased from Sigma-Aldrich (St-Louis, MO). The 2 lentivirus shRNA constructs used for FABP7 depletion in A4-007 have been previously described.^18^ The 4 lentivirus PRKCA shRNA constructs (The RNAi Consortium shRNA Library, Broad Institute) were obtained from the University of Alberta RNAi Core Facility (see Supplementary Table 1). The MISSION pLKO.1 plasmid (SHC002; Sigma-Aldrich) served as a control.

### Patient-Derived GBM Xenografts

Animal experiments were reviewed and approved by the Cross Cancer Institute Animal Care Committee under protocols AC19249 and AC23271. All animal experiments were in accordance with the Canadian Council on Animal Care guidelines. Patient-derived neurosphere cells were injected intracranially into the brains of immunodeficient 8-week-old male NOD.Cg-PrkdcscidIl2rg (NSG) mice as previously.^19^ 100 000 cells (in 5 μL) were injected into the right frontal cortex at a depth of 2 mm (1.5 mm lateral, 1 mm anterior from the bregma). See Supplementary Methods for protocols used to test the effects of FABP7 inhibition and radiation treatment on mouse survival using an orthotopic model of GBM.

### Immunohistochemistry, Immunofluorescence, and Transmission Electron Microscopy

Tissues from GBM patient tumors and mouse brain tumors were processed and immunostained as previously described.^16,20^ For immunostaining of patient-derived GBM neurosphere cells, cells were cultured, processed, and imaged directly on 96-well plates (Greiner Bio-One, Kremsmünster, Austria). Tumor tissues and GBM neurosphere cells were immunostained with anti-FABP7 antibody (Santa Cruz Biotechnology, Dallas, Texas), human-specific anti-nestin antibody (Abcam, Cambridge, UK), anti-GAP43 antibody (Abcam), anti-GAP43-pSer41 (Sigma-Aldrich), and anti-Connexin 43 (Cx43) antibody (Cell Signaling Technology, Danvers, MA). For immunohistochemistry, the Dako Cytomation EnVision + secondary system (Dako, CA) was used to visualize the primary antibody. Tissues were counterstained with hematoxylin. For immunofluorescence, Alexa-555 and Alexa-647 secondary antibodies (Invitrogen) were used to visualize the signal. Images were acquired using a brightfield microscope for immunohistochemistry and a Zeiss confocal microscope for immunofluorescence microscopy. See Supplementary Methods for transmission electron microscopy analysis of TMs in GBM neurosphere cultures.

### Cell Viability Assays

GBM cells (50 000 cells/per 0.32 cm^2^ well; 96-well plates) were treated with TMZ (0–1000 μM). After 5 days, adherent cells were fixed and stained with 0.5% crystal violet as previously described.^21^ For the rezasurin assay, control and SBFI-26-treated GBM cells (20 µM) (14 000 cells/well) were treated with TMZ (0–2000 µM). After 5 days, the medium was removed and cells were incubated in 20 µL (3 µg) rezasurin for 2 h at 37 °C and fluorescence measured using a 544 nm excitation/590 nm emission filter set (FLUOstar OPTIMA microplate reader; BMG Labtech, Ortenberg, Germany).

### Semiquantitative and Quantitative RT-PCR

Total RNA was isolated from GBM cell lines as previously described using TRIzol (Thermo Fisher Scientific).^18^ cDNA was generated using Superscript II reverse transcriptase (Life Technologies, Carlsbad, CA) and oligo dT (semiquantitative RT-PCR or random primers [RT-qPCR]) (see Supplementary Table 1 for list of primers). QuantStudio6 real-time PCR system (Applied Biosystems, Waltham, MA) and SYBR Green Master Mix (Applied Biological Materials Inc.) were used to carry out RT-qPCR reactions.

### Western Blot Analysis

Whole-cell protein lysates were prepared as previously described.^22^ Forty μg protein lysates were loaded and resolved on SDS-PAGE gels, transferred to nitrocellulose membranes and immunoblotted with mouse anti-FABP7 (1:500, Santa Cruz Biotechnology), rabbit anti-GAP43 (1:2000, Abcam), rabbit anti-pGAP43 (1:500, Sigma-Aldrich), rabbit anti-PKCα (1:5000, Cell Signaling Technology) or mouse anti-GAPDH (1:1000, Thermo Fisher Scientific) antibodies, followed by horseradish peroxidase-conjugated secondary antibodies (1:25000, Thermo Fisher Scientific). Signals were visualized by ECL Western Detection Reagent (GE Healthcare Life Sciences) or SuperSignal West Pico PLUS (Thermo Fisher Scientific).

### TM Quantification Analysis

Images of live GBM cells were taken with a Zeiss Axiovert 200M LMP microscope using a 10X lens with Metamorph software (Version 7.8, Molecular Devices, Sunnyvale, CA, USA). Ten to twenty separate frames were captured for each experimental group. Image analysis of TM formation upon FABP7 inhibition and PKC inhibition in A4-007 and A4-016 was performed in MATLAB (MathWorks).^23^ Image analyses of TM formation in A4-007 control and *PRKCA* knockdown cells were performed using MetaXpress (Version 6.5, Molecular Devices, Sunnyvale, CA, USA). See Supplementary Methods for details.

### Live Cell Imaging—Single-Cell Tracking and Calcium Flux

Time-lapse video microscopy was performed on GBM neurosphere cells to track cell migration along TMs and calcium flux. For cell tracking along TMs, GBM neurosphere cells were plated in 24-well plates and cultured for 120 h with continuous microscopic tracking. Ten to fifteen different stage positions for each experimental group were set to acquire images every hour. In each experiment, the movement of more than 50 individual cells was tracked using Manual Tracking plug-in of ImageJ software. The directionality and the velocity of cell movement were measured using the Chemotaxis and Migration Tool plug-in (ibidi) of ImageJ software. Calcium signal spread upon laser injury was measured using FLUO-4 AM^24,25^ (see Supplementary Methods).

### Transwell Assay

The Transwell migration assay was used to measure directional cell migration as previously described.^18^ One hundred thousand cells were seeded in the top chambers of 24-well Transwell inserts (8 µm) in neurosphere medium with or without SBFI-26. Cells in the top chambers were allowed to migrate towards the bottom chamber containing DMEM with 10% fetal calf serum (chemoattractant) over 24 h or 48 h. Cells in the bottom chamber were fixed, stained with crystal violet, and counted as described previously.^18^

### Databases and Statistical Analysis

Correlation between *FABP7*, *GAP43*, and *TTYH1* RNA levels in human GBM tissues were examined using GBM patient cohorts available through cBioportal (www.cbioportal.org/datasets) and a single-cell sequencing dataset (www.gbmseq.org).^26^ The prognostic significance of *FABP7* and *GAP43* RNA levels in GBM patients was tested using the TCGA HG-U133A gene expression dataset (453 GBM tissues). The statistical significance of Kaplan–Meier survival plot and the Pearson correlation coefficient were examined using MedCalc Software. The statistical significance of 2 experimental group comparisons was examined using a 2-sided unpaired Student’s *t*-test. See Supplementary Methods for details regarding the study design for survival experiments using an orthotopic mouse model of glioblastoma.

## Results

### FABP7 Is Abundantly Expressed in GBM TM-Like Fibers

Tumor tissue was obtained from patients diagnosed with GBM (Supplementary Table 2) and embedded in paraffin. FABP7 immunostaining of GBM tissue sections revealed FABP7 in both the nucleus and cytoplasm of a subset of tumor cells, as well as in very long fibers reminiscent of TMs emanating from GBM cells (Supplementary Figure 1A; patients A4-007, A4-009, A4-012). We next examined whether TM-like protrusions were also present in NSG mice intracranially injected with neurosphere cultures derived from 3 patients (A4-007, A4-012, and ED511). We confirmed the presence of FABP7-enriched ultra-long fibers in the tumor regions of mouse brains (Supplementary Figure 1B). As nestin has been extensively used to visualize TMs in GBM tumors,^4,24^ we carried out co-immunostaining analysis with FABP7 and nestin antibodies. We found co-localization of FABP7 and nestin in GBM fibers located in the brains of mice intracranially injected with A4-007 neurosphere cells (Supplementary Figure 1C). Upon closer examination, we observed nestin-positive fibers in both the cores and infiltrative zones of orthotopic tumors (Supplementary Figure 1D), whereas FABP7 was primarily found in fibers originating from cells located at infiltrative zones (Supplementary Figure 1E).

### TMs Serve as Conduits for Rapid Directional GBM Cell Migration

**Figure 1.**
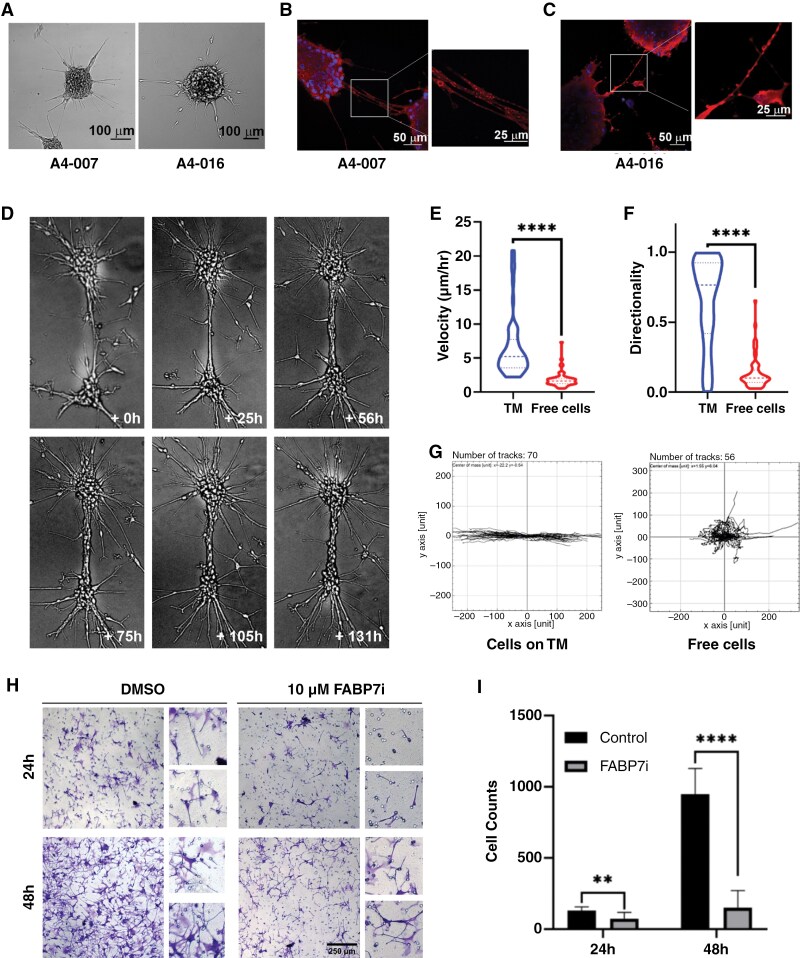
FABP7 is associated with infiltrative TMs which function as structural fibers for directional cell movement. (A) Bright field images showing TM formation in A4-007 and A4-016 cells cultured under neurosphere conditions. Scale bar = 100 µm. (B and C) Representative immunofluorescence images showing FABP7 expression in the TMs of A4-007 (B) and A4-016 neurospheres (C). Scale bars = 50 µm and 25 µm. (D) Representative bright field time-lapse images showing A4-018 GBM cells migrating along TMs. (E and F) Analysis of time-lapse images showing the difference in velocity (E) and directionality (F) of cells migrating along TMs vs non-TM-containing “free” cells. More than fifty individual cells were tracked in each group for analysis. Images were acquired every hour for 120 h. (G) Trajectory plots showing the paths of individual cells when cells are on TMs vs free cells. (H) Representative images of Transwell migration assays comparing the migration of A4-018 cells with or without FABP7 inhibition at 24 h and 48 h after plating cells in the upper chamber. Magnified images are located on the right. Scale bar = 250 µm. (I) Histograms showing numbers of migrated cells in Transwells plated with control cells and cells treated with FABP7 inhibitor SBFI-26 (FABP7i).

A subset of GBM tumors cultured under neurosphere conditions forms stable ultra-long fibers in vitro (Figure 1A). In this study, we focused on 3 patient-derived GBM cell lines: A4-007 with a doubling time of 1 week, A4-016 with a doubling time of 2 weeks and A4-018 with a doubling time of 3 weeks. GBM neurosphere fibers express abundant FABP7 (A4-007 shown in Figure 1B and A4-016 shown in Figure 1C). Three approaches were used to functionally characterize the ultra-long fibers formed by GBM neurospheres in culture. First, we used the calcium signaling probe FLUO-4 AM to demonstrate Ca^2+^ flux across fibers in A4-007 GBM neurosphere cultures (Supplementary Video 1 and Supplementary Figure 2A). Second, transmission electron micrographs of fibers from A4-018 GBM neurosphere cultures revealed the presence of robust mitochondria and endoplasmic reticulum, along with extensive microtubule assembly (Supplementary Figure 2B). Third, immunostaining analysis of the gap junction protein, Connexin 43 (Cx43), indicative of intercellular communication, revealed abundant expression in A4-007 GBM neurospheres and fibers (Supplementary Figure 2C). Therefore, fibers observed in GBM neurosphere cultures fulfill the criteria of functional TMs with roles in intercellular communication as previously defined.^4,6,27,28^

We next asked whether TMs might be providing structural support for directional migration of GBM cells. We used live cell time-lapse imaging to study the migration of cells in GBM neurosphere cultures. We found abundant examples of A4-018 GBM cells migrating along TMs linking neurospheres to one another, with TMs serving as highly efficient cell migration networks (Figure 1D; Supplementary Video 2). Similar observations were made in A4-016 GBM neurosphere cultures (Supplementary Figure 3). Although migrations were mostly unidirectional, we also observed cells reversing their direction, suggesting complex responses to migratory cues. We then analyzed the speed and directionality of cells traveling along TMs vs the speed and directionality of cell movements in the absence of TMs. Cells traveling along TMs moved significantly faster compared to TM-free cells (average of 6.5 μm/h on TMs vs 1.8 μm/h for TM-free cells; Figure 1E). Furthermore, the movement of cells along TMs was more directional compared to free cells, in keeping with TMs guiding cell migration (Directionality score of 0.65 on TMs vs 0.14 of TM-free cells; Figure 1F and G).

We also used the Transwell migration assay to examine the migration of TM-forming GBM neurosphere cultures. Remarkably, A4-018 cells showed TMs emanating from the pores on the bottom side of the Transwell membrane ahead of the cells attached to them. These results are in keeping with TMs serving as structural guides for directional cell migration. As FABP7 is expressed in TM-forming GBM neurosphere and FABP7 has previously been shown to promote cell migration, we tested the effect of FABP7 inhibition on A4-018 migration. Fewer TMs at the bottom of Transwell chambers as well as fewer migrating cells were observed upon inhibition of FABP7 with SBFI-26 (Figure 1H and I), suggesting that FABP7 promotes TM-guided migration of GBM cells.

### FABP7 and GAP43 Levels Are Significantly Correlated in GBM Tumors

**Figure 2.**
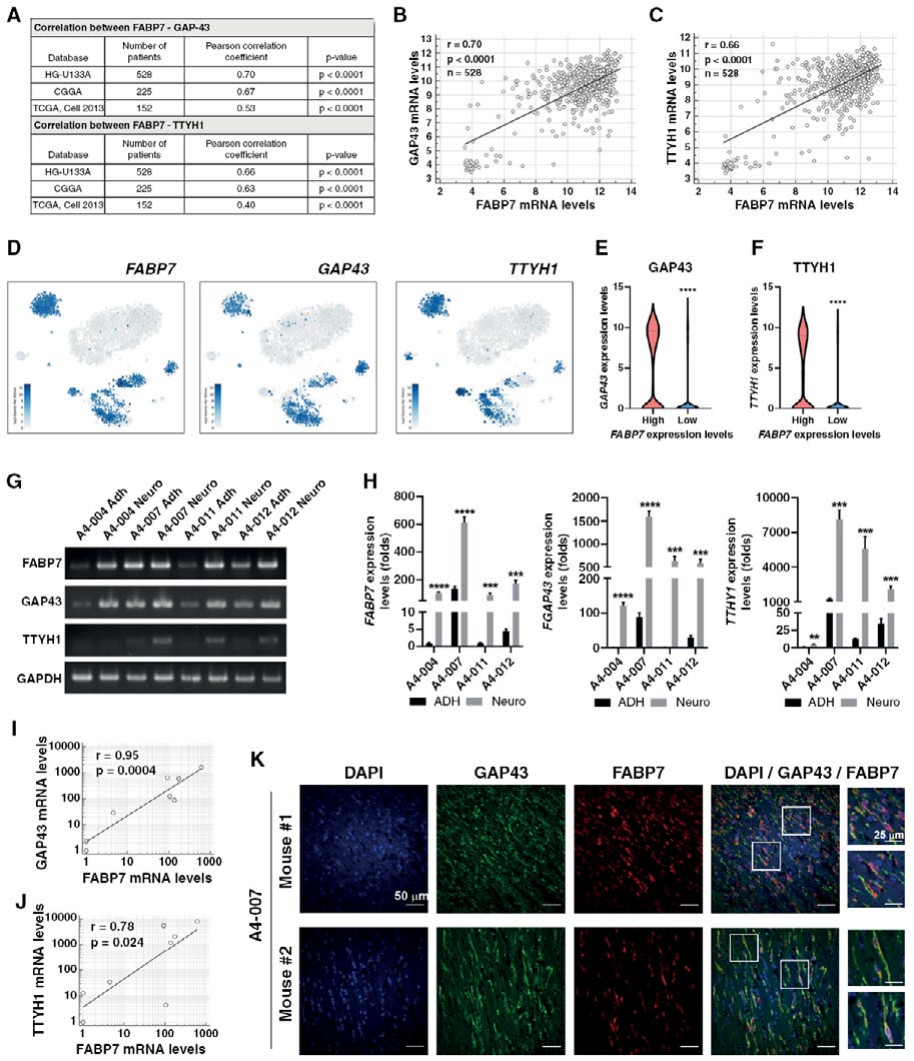
FABP7 and TM markers are significantly correlated. (A) Summary of correlations between *FABP7* and TM markers (*GAP43* and *TTYH1*) in 3 independent TCGA datasets (HG-U133A, CGGA, and TCGA Cell 2013), with all 3 datasets showing strong correlation. (B and C) *GAP43* (B) or *TTYH1* (C) RNA levels show significant correlation with *FABP7* in a TCGA gene profiling dataset (HG-U133A). (D) UMAP depicting *FABP7*, *GAP43*, and *TTYH1* expressing cells in GBM cell clusters generated from a GBM single-cell sequencing dataset.^26^ (E and F) Analysis of scRNA-seq data showing significantly higher RNA levels of *GAP43* (E) and *TTYH1* (F) in cells with high FABP7 using the median value as a cutoff. (G) Semiquantitative RT-PCR analysis and RT-qPCR analysis (H) of *FABP7*, *GAP43*, and *TTYH1* RNA levels in paired patient-derived GBM neurosphere cells (Neuro) and their differentiated counterparts (Adh). (I and J) *FABP7* and either *GAP43* (I) or *TTYH1* (J) RNA levels in a panel of patient-derived GBM cell lines and their differentiated counterparts showed significant correlation. The correlation plots were generated from RT-qPCR analysis. (K) A4-007 mouse xenograft tumor tissues were co-immunostained with FABP7 and GAP43 antibodies. The area in square is magnified on the right. Scale bars = 50 µm or 25 µm as indicated.

GAP43 knockdown results in reduced formation of TMs,^4,5^ and TTYH1 depletion reduces TM formation and invasion speed,^29^ suggesting that GAP43 and TTYH1 are important drivers of TM formation. We first asked whether there was a correlation between *FABP7* RNA levels and that of *GAP43* and *TTYH1* using TCGA bulk RNA-seq datasets from GBM tumor tissues. *FABP7* RNA levels showed a strong positive correlation with both *GAP43* (R-value varies from 0.53 to 0.70, *P* < .0001) and *TTYH1* RNA levels (*R*-value varies from 0.40 to 0.66, *P* < .0001) (Figure 2A–C). To further examine whether these genes are correlated at the single-cell level, we used a single-cell GBM RNA-seq dataset. We found that *FABP7*, *GAP43*, and *TTYH1* RNAs are all present in the same clusters (Figure 2D). When we divided the cells into *FABP7*-high vs *FABP7*-low cells using the median value as cutoff, FABP7-high cells had significantly higher levels of *GAP43* (Figure 2E) and *TTYH1* compared to FABP7-low cells (Figure 2F).

We examined levels of *FABP7*, *GAP43*, and *TTYH1* RNA in paired patient-derived GBM cultures: those cultured under neurosphere conditions (Neuro) and those cultured under standard FCS-containing medium known to promote GBM differentiation (Adh) using semiquantitative RT-PCR (Figure 2G) and quantitative RT-qPCR (Figure 2H). Consistent with patient data, we found that *FABP7* RNA levels were strongly correlated with that of *GAP43* (Figure 2I; *r* = 0.95, *P* = .0004) and *TTYH1* (Figure 2J; *r* = 0.78, *P* = .024). Similar to *FABP7*, both *GAP43* and *TTYH1* mRNAs were present at much higher levels in GBM cells cultured under neurosphere conditions compared to their differentiated counterparts, confirming the correlation between TM formation and culture conditions that promote stemness.^30,31^ We next used co-immunostaining analysis to examine the correlation between GAP43 and FABP7 in the brains of mice orthotopically injected with A4-007 neurosphere cells. We observed extensive co-immunostaining of these 2 proteins in GBMs, but especially in TMs (Figure 2K), suggesting the possibility of a functional link between FABP7 and GAP43.

Patients with high levels of *GAP43* RNA have a significantly worse prognosis compared to those with low levels of *GAP43* RNA (Supplementary Figure 4A; HR = 1.45, *P* = .0001). Importantly, when we further divided patient populations into those with high versus those with low levels of *FABP7* RNA, we found no significant difference between *GAP43* high compared to *GAP43* low in the population with low levels of FABP7 (Supplementary Figure 4B; HR = 1.16, *P* = .42), while retaining significant difference in patients with high levels of FABP7 (Supplementary Figure 4C; HR = 1.48, *P* = .001). This suggests that GAP43-mediated GBM aggressiveness is dependent on high levels of FABP7. Further investigation of the prognostic significance of *FABP7* and *GAP43* as a function of age revealed a highly significant correlation between elevated *FABP7* and *GAP43* RNA levels and lower survival probability in patients ≤60 years of age, with patients ≤40 years of age being particularly susceptible to elevated levels of *FABP7* (Supplementary Figures 5 and 6). Neither *FABP7* nor *GAP43* levels showed a differential prognostic significance as a function of patient sex. Together, our data point to a functional link between FABP7 and GAP43 expression, with high levels of FABP7 associated with decreased survival in GBM patients, particularly in younger patients.

### FABP7 Promotes TM Formation Through GAP43 Phosphorylation

**Figure 3.**
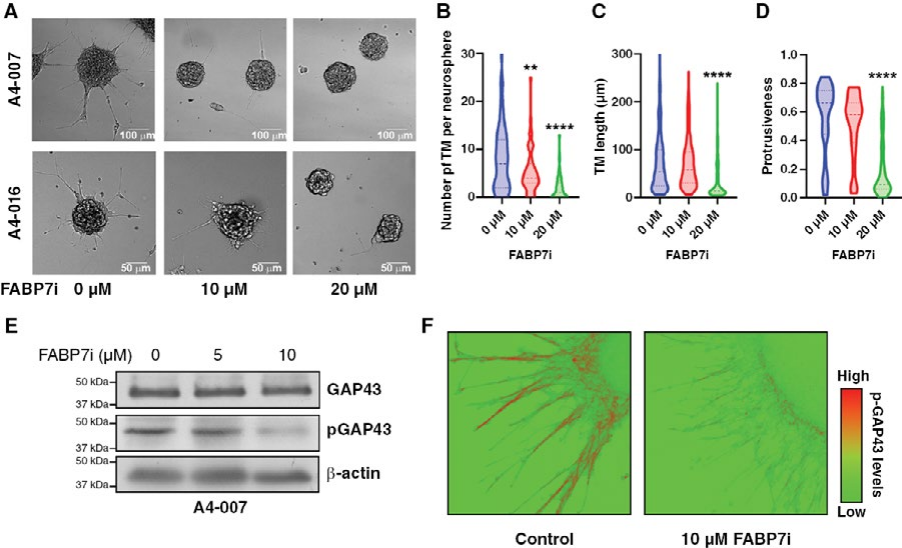
FABP7 promotes TM formation and GAP43 phosphorylation. (A) Representative bright field images showing decreased TM formation upon FABP7 inhibition in A4-007 and A4-016 cells. Scale bars = 100 µm or 50 µm as indicated. (B–D) 10–20 images (1–4 neurospheres per image) were taken for each condition and images were analyzed for the number of TMs per neurosphere (B), TM length (C), and protrusiveness (D). (E) Western blot analysis showing decreased pGAP43 levels upon FABP7 inhibition in A4-007 cells. (F) Representative immunofluorescence images showing changes in levels (low—green and high—red) and distribution of pGAP43 upon FABP7 inhibition in A4-018 cells.

We next addressed a possible role for FABP7 in GBM TM formation using a small molecule inhibitor of FABP7, SBFI-26. We observed strong suppression of TM formation upon FABP7 inhibition in A4-007 and A4-016 cells (Figure 3A). When we analyzed images (ten to twenty images per treatment group), there was a dose-dependent decrease in the numbers of TM per neurosphere (Figure 3B), TM length (Figure 3C), as well as protrusiveness (Figure 3D). To address potential off-target effects of the inhibitor, we knocked down FABP7 in A4-007 neurosphere cultures using shRNA to evaluate TM formation. Consistent with FABP7 inhibition, we observed an extensive reduction in TM formation upon FABP7 knockdown (Supplementary Figure 7).

GAP43 phosphorylation at S41 is required for its activity during brain development, with roles in the formation of neuronal protrusions and neuronal growth cones.^32^ AA, the ligand of FABP7 that is upregulated relative to docosahexaenoic acid (DHA) in the GBM microenvironment,^33^ has been shown to facilitate GAP43 phosphorylation through PKC activation, resulting in increased neuronal protrusions.^8^ Recently, phosphoproteomic mass spectrometry analysis of GBM cells with TMs revealed upregulation of GAP43-pS41,^34^ suggesting that phosphorylation of GAP43 may be an important regulatory event in TM formation. As AA activates PKC, which in turn phosphorylates GAP43, we hypothesized that FABP7 may be instrumental to GAP43 activation and TM formation by facilitating AA uptake from the AA-rich GBM tumor microenvironment and making AA accessible to PKC. We first explored the possibility that FABP7 regulates TM formation by modulating levels of GAP43 and/or pGAP43. Upon inhibition of FABP7 in A4-007 GBM neurosphere cultures, we found a decrease in GAP43 phosphorylation with GAP43 expression levels remaining constant (Figure 3E). Next, we examined the localization of pGAP43 in A4-018 neurosphere cultures by immunofluorescence analysis. We observed high levels of pGAP43 on the outer edges of neurospheres and TMs (Figure 3F). When these cells were treated with the FABP7 inhibitor, SBFI-26, levels of pGAP43 significantly decreased (Figure 3F). When combined with the dramatic reduction in TM formation observed upon FABP7 inhibition, these results suggest that FABP7 regulates GAP43 phosphorylation with GAP43 phosphorylation being the key to robust TM formation.

### FABP7 Promotes GAP43 Phosphorylation Through PKC Activation

**Figure 4.**
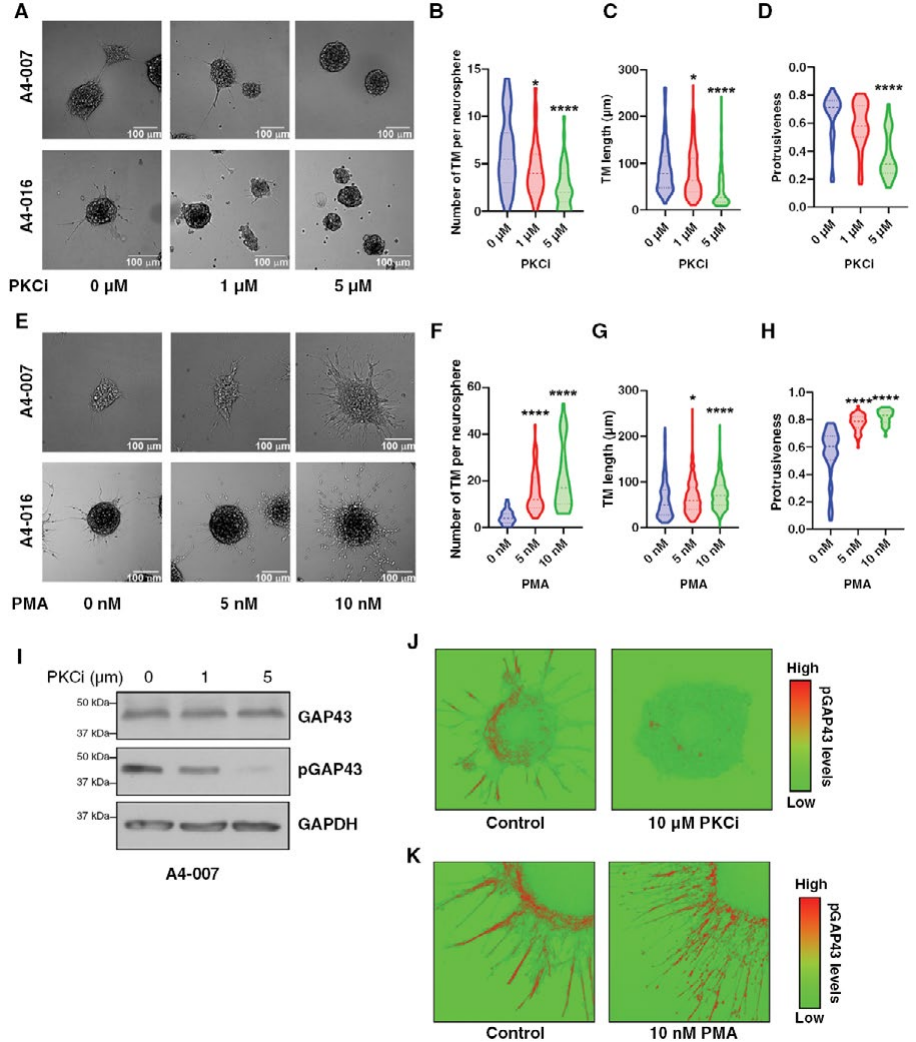
PKC promotes TM formation and GAP43 phosphorylation. (A) Representative bright field images showing decreased TM formation upon PKC inhibition in A4-007 and A4-016 cells. Scale bars = 100 µm. (B–D) 10–20 images (1–4 neurospheres per image) were taken for each condition and images were analyzed for number of TM per neurosphere (B), TM length (C), and protrusiveness (D). (E) Representative bright field images showing increased TM formation upon PKC activation using PMA in A4-007 and A4-016 cells. Scale bars = 100 µm. (F–H) 10–20 images were taken for each condition and images were then analyzed for the number of TM per neurosphere (F), TM length (G), and protrusiveness (H). (I) Western blot analysis showing decreased pGAP43 levels upon PKC inhibition in A4-007 cells. (J) Representative immunofluorescence images of A4-018 cells showing changes in levels and distribution of pGAP43 upon PKC inhibition. (K) Representative immunofluorescence images of A4-018 cells showing changes in levels and distribution of pGAP43 upon PKC activation by PMA.

While PKC has been shown to phosphorylate and activate GAP43, and AA is essential for PKC activation in neuronal cells,^8,9^ it is still unclear whether GAP43 is a substrate of PKC in GBM cells. FABP7, an intracellular trafficker of AA, has been shown to modulate PKC activity in GBM cells.^35^ We therefore asked whether FABP7 might regulate GAP43 phosphorylation through PKC activation. We first used the PKC inhibitor (PKCi), enzastaurin, to attenuate PKC activity. Enzastaurin is a potent inhibitor of PKCβ (IC50 = 6 nM in cell-free assays), which also shows selectivity towards PKCα (IC50 = 39 Nm) (SelleckChem.com). We observed strong suppression of TM formation upon inhibition of PKC activity in A4-007 and A4-016 neurospheres (Figure 4A). Detailed analysis of TMs across 10 to 20 images per group revealed decreased numbers of TMs per neurosphere (Figure 4B), decreased TM length (Figure 4C), and decreased protrusiveness (Figure 4D). When we repeated the experiment with the PKC activator, phorbol 12-myristate 13-acetate (PMA), we observed the opposite effect, with dramatically increased TM formation in A4-007 and A4-016 neurospheres (Figure 4E). Dose-dependent increases in the numbers of TM (Figure 4F), TM length (Figure 4G), and protrusiveness (Figure 4H) were observed upon analysis of TMs found in A4-007 neurosphere cultures.

Next, we examined GAP43 phosphorylation upon PKC inhibition or PMA treatment in A4-007 cells. We observed a dose-dependent decrease in pGAP43 levels upon PKC inhibition, with no change in overall GAP43 levels (Figure 4I). Immunofluorescence analysis of A4-018 cells showed abundant expression of pGAP43 at the outer edges of neurospheres and TMs (Figure 4J). PKC inhibition resulted in decreased pGAP43 levels (Figure 4J), whereas PKC activation by PMA increased pGAP43 expression in TMs (Figure 4K).

To further investigate the importance of PKC in TM formation and GAP43 phosphorylation, we examined the expression profiles of *PRKCA* and *PRKCB* in TM-forming GBM neurosphere cultures along with one GBM neurosphere culture unable to form TMs (A4-004), one GBM neurosphere culture that lost the ability to form TMs (A4-012) and one adherent cell line (A4-004 cultured in medium supplemented with fetal calf serum). RT-qPCR analysis revealed elevated levels of *FABP7* and *GAP43* RNA in all GBM neurospheres tested (Supplementary Figure 8), with an ~5X reduction in *FABP7* RNA levels in non-TM-forming A4-012 compared to TM-forming A4-012 cells. *PRKCA* RNA levels were high in all samples except for non-TM-forming A4-012 cells. *PRKCB* RNA was not detected in our GBM neurosphere cultures with the exception of A4-012 (TM and non-TM forming) and A4-018.

**Figure 5.**
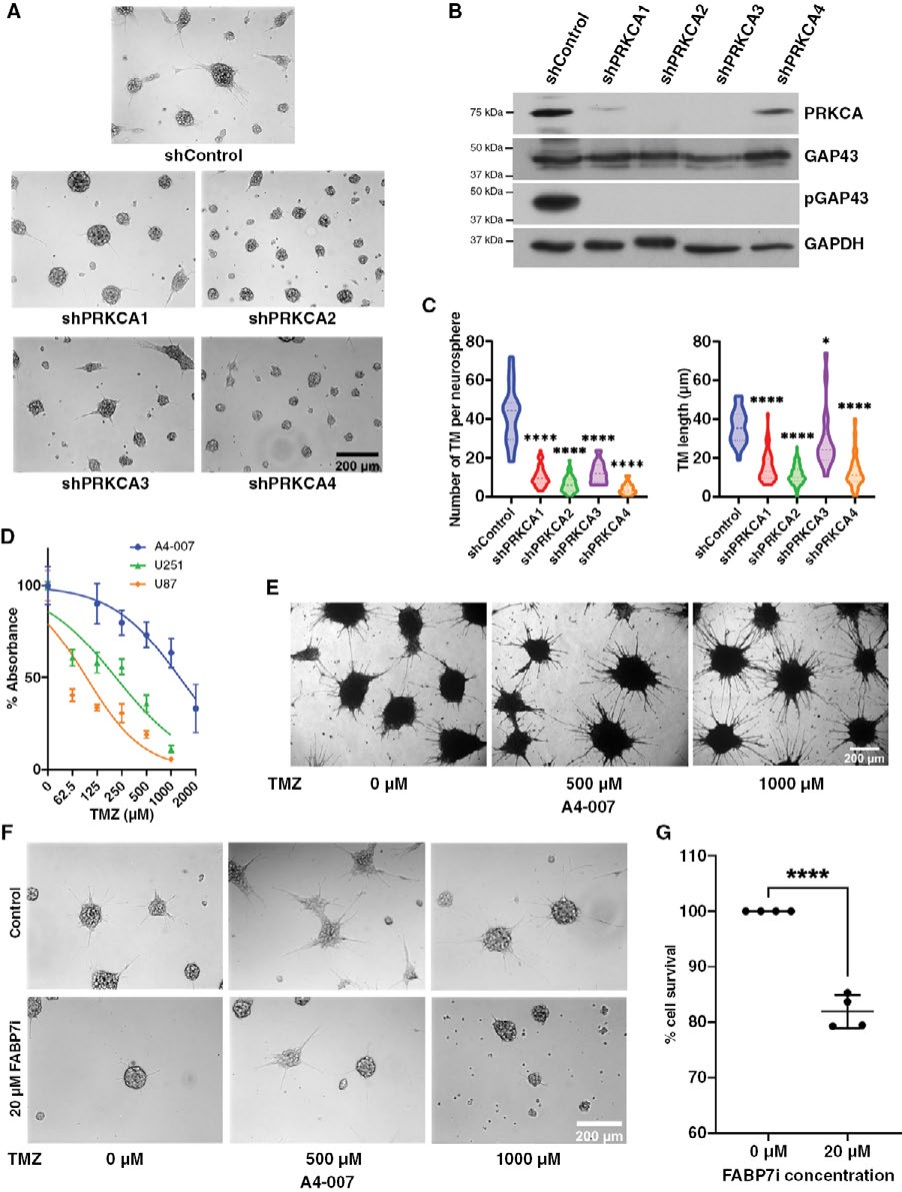
PRKCA knockdown inhibits TM formation (A–C) and FABP7 expression increases TM formation in temozolomide-treated cells (D–G). (A) Representative bright field images showing decreased TM formation in A4-007 GBM neurosphere cultures infected with 4 lentivirus vectors containing different shRNAs targeting *PRKCA* compared to cells infected with a control lentivirus vector. Scale bar = 200 µm. (B) Western blot of lysates prepared from control and PRKCA-targeted A4-007 GBM neurosphere cultures using antibodies to PKCα, GAP43, pGAP43, and GAPDH. (C) Ten to twenty bright field images taken from each of the 5 infected cultures (control and 4 PRKCA knockdowns) were analyzed for the number of TMs per neurosphere and the length of TMs. (D) The crystal violet assay was used to measure viable A4-007, U87, and U251 cells treated with vehicle or TMZ for 5 days. (E) Representative images showing increased TM formation in crystal violet-stained A4-007 GBM neurosphere cultures as a function of increasing concentrations of TMZ after 5 days of treatment. Cells were seeded at 50 000/well (150 000/cm^2^). Scale bar = 200 µm. (F) Representative bright field images showing increased TM formation in control A4-007 cultures treated with TMZ, and attenuated TM formation in TMZ-treated cultures. Cells were seeded at 50 000/cm^2^. Scale bar = 200 µm. (G) The rezasurin assay was used to measure cell viability in A4-007 control and FABP7-inhibited GBM neurosphere cultures. Each filled circle represents an independent experiment, with cell survival in control cells set at 100% for each experiment.

We next used 4 lentivirus shRNA constructs targeting different regions of *PRKCA* to infect A4-007 neurosphere cultures. All 4 *PRKCA* shRNA constructs effectively reduced PKCα expression in A4-007 cells compared to cells infected with control lentivirus vector (Figure 5A). While GAP43 levels were not affected by *PRKCA* knockdown, pGAP43 was undetectable in PKCα-depleted cells (Figure 5B). PKCα-knockdown resulted in dramatically reduced TM numbers and TM lengths in the case of shPRKCA1, shPRKCA2, and shPRKCA4, with significant reduction also observed for shPRKCA3 (Figure 5A and C). Our combined results implicate PKCα in GAP43 phosphorylation and TM formation, and suggest that PKC activation resides downstream of FABP7.

### Targeting FABP7 Attenuates Temozolomide-Induced TM Formation

TM-connected GBM stem-like cells have been shown to resist TMZ treatment with increased cell survival in vivo.^5^ TMZ treatment also increased TM length in a human organotypic slice culture injected with patient-derived GBM cells.^36^ We therefore asked whether FABP7 plays a role in the survival of TMZ-treated GBM neurosphere cultures. Using the crystal violet cell survival assay, we found that TM-forming FABP7-positive A4-007 cells were more resistant to TMZ treatment than FABP7-negative U87 and FABP7-positive U251 adherent cells (Figure 5D). In support of the importance of TMs in chemoresistance, we observed a marked increase in TM formation in A4-007 GBM neurosphere cultures upon treatment with TMZ [crystal violet stained; 150 000 cells/cm^2^ (Figure 5E) and bright field microscopy; 50 000 cells/cm^2^ (Figure 5F, top panel)]. FABP7-inhibition attenuated TM formation in TMZ-treated cultures (Figure 5F, bottom panel). The reduced number of neurospheres observed upon SBFI-26 treatment of A4-007 cultures in the absence of TMZ is in keeping with the 15%–20% decrease in cell viability observed using the rezasurin cell viability assay (Figure 5G).

### FABP7 Inhibition Increases Survival in an Orthotopic Mouse Brain GBM Model

**Figure 6.**
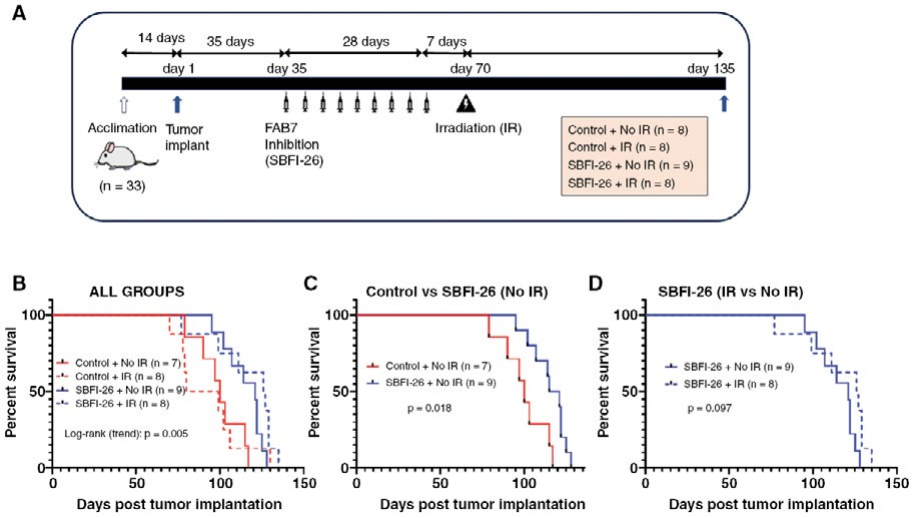
FABP7 inhibitor SBFI-26 extends survival in an orthotopic mouse model of TM-forming GBM cells. (A) TM-forming A4-007 neurosphere cells were intracranially implanted into NSG mice (*n* = 33) (day 1). Starting on day 35 after tumor implantation, mice were intraperitoneally injected with vehicle control (*n* = 16) or FABP7 inhibitor, SBFI-26 (*n* = 17). Intraperitoneal injections were given every 3–4 days (for a total of 9 injections) with the final injection given on day 63. One week after the last intraperitoneal injection (on day 70), half the mice from the vehicle control and SBFI-26 groups were given a single dose of 800 cGy radiation using SARRP. The experiment was terminated at 135 days post-tumor implantation. One mouse from the control group that survived past 135 days showed no sign of weight loss or poor health and was not included in the analysis. (B–D) Kaplan–Meier survival plots showing survival distributions of experimental animal groups. Statistical significance of survival trend among all 4 groups (B) or differences between 2 groups (C and D) were analyzed using log-rank test and indicated by *P*-values. “*n*” denotes sample size; IR, irradiation.

To address whether FABP7 inhibition can prolong survival in vivo, we injected TM-forming FABP7-positive A4-007 cells into the brains of NSG mice. Tumors were allowed to form over a period of 5 weeks before mice were intraperitoneally injected with either vehicle control or FABP7 inhibitor (SBFI-26) twice weekly (9 injections in total) (Figure 6A). The brains of half of the mice in vehicle and SBFI-26 groups were then irradiated to assess induced changes in radiosensitivity. A single dose of 800 cGy was administered in a single fraction using the Small Animal Radiation Research Platform. Experimental endpoints were based on weight loss, poor health, or 135 days after tumor injection.

Statistical analysis of the whole population showed an overall effect on survival based on treatment (Figure 6B). A significant difference in survival was observed between “Control – No Radiation” and “Inhibitor – No Radiation” groups (Figure 6C). Mice treated with SBFI-26 survived 15 days longer on average than control mice (115 days vs 100 days; *P* = .03). There was no significant difference in survival between “SBFI-26 – No Radiation” and “SBFI-26 – Radiation” groups, suggesting that SBFI-26 treatment did not sensitize the tumor to radiation (Figure 6D).

## Discussion

The highly infiltrative nature of GBM and its resistance to therapy are significant obstacles to improving patient outcomes.^3^ TMs, ultra-long membrane protrusions, have been linked to both GBM cell infiltration and therapy resistance in GBM tumors, suggesting that targeting TMs may benefit the clinical management of these treatment-recalcitrant tumors.^6,27,37^ TMs interconnect GBM-GBM and GBM-normal brain cells, forming cellular networks believed to promote tumor survival when patients are treated with radiotherapy and chemotherapy.^37^ The striking increase in TM formation that we and others^5^ have observed in GBM stem-like cells upon TMZ treatment and accompanying resistance to TMZ strongly support a role for TMs in therapy resistance.

A subpopulation of TMs (called invasive TMs) was recently identified, with the tips of these invasive TMs resembling the growth cones of neuronal protrusions, which are important for pathfinding in migrating neurons.^37,38^ These TMs facilitate GBM cell movement through branching, protrusion, and retraction, common neuronal migration mechanisms during neurodevelopment. Here, we identify TMs that appear to function as a scaffold for the rapid and directional migration of GBM cells (Supplementary Figure 9). Our findings resonate with recent findings showing spatial organizations within GBM tumors that resemble those found in the developing brain.^39,40^ In particular, radial glial fibers provide tracts for directional movement of neuroblasts in both the developing brain and towards sites of injury in the neonatal brain.^41,42^ Analogous to radial glial fibers, TMs directed towards sites of surgical resection have been observed in mouse models of GBM.^5^ TM formation may thus explain why a high percentage of GBM tumors recur within 2 cm of the resected tumor margins.^43^ Together, these data point to key roles for TMs in tumor recurrence and highlight the need for identifying molecular mechanisms driving TM formation.

GBM tumors and brain development share many features,^38^ including expression of radial glial cell marker FABP7 and neuronal markers such as GAP43, formation of processes and fiber-like structures, cellular hierarchies, and spatial organizations.^39,40^ Molecular drivers of TM formation, such as GAP43 and TTYH1, play important roles in neurite outgrowth and neurodevelopment. In particular, GAP43 regulates growth cone stability by promoting actin polymerization.^44^ GAP43 is phosphorylated at Serine-41 by PKC and dephosphorylated by the calcineurin phosphatase.^45,46^ AA, a preferred ligand of FABP7, activates PKC to selectively facilitate the phosphorylation of GAP43 in neuronal cells.^8,9^ We postulate that expression of FABP7 in the GBM AA-rich environment^33,47^ facilitates AA-dependent activation of PKC, with subsequent phosphorylation of GAP43 at S41, which then promotes fiber formation (Supplementary Figure 9). GAP43 phosphorylation at sites S41 and S154 has been identified as significantly upregulated phosphorylation sites in cells with abundant TMs using phosphoproteomic mass spectrometry, further supporting our model.^34^

Our data indicate that there is a significant correlation between *FABP7* and *GAP43* RNA levels in both bulk tumors and at the single-cell level using high-throughput RNA sequencing. Interestingly, high levels of *FABP7* or *GAP43* RNA are associated with poor clinical outcomes, especially in young (≤40 years) GBM patients. However, the prognostic significance of elevated *GAP43* becomes negligible when *FABP7* levels are low. These results suggest that FABP7 may reside upstream of GAP43 activation, in keeping with our model. Inhibition of FABP7 results in reduced phosphorylation of GAP43 and reduced TM formation, suggesting that FABP7 promotes TM formation by facilitating phosphorylation of GAP43. Using the PKC inhibitor, enzastaurin and the PKC activator, PMA, we showed that PKC is indeed responsible for GAP43 phosphorylation in GBM cells. Our results further indicate that PKCα is widely expressed in our TM-forming GBM neurosphere cultures and that depletion of PKCα results in inhibition of GAP43 phosphorylation and TM formation. Therefore, we propose that TM formation in GBM cells is mediated through an FABP7-PKC-pGAP43 axis.

Our results indicate that FABP7 inhibition extends the survival of mice that have been intracranially injected with TM-forming GBM cells. SBFI-26-treated mice survived an average of 15 days longer than control mice, with 5/9 mice treated with SBFI-26 surviving more than 120 days. As SBFI-26 treatment ended 63 days after tumor injection, it is possible that survival could have been extended even longer with continuous SBFI-26 treatment. Combining SBFI-26 with radiation did not further extend mouse survival, suggesting that survival benefits are entirely dependent on SBFI-26 treatment. In light of our observation that TMZ enhances formation of TMs and pharmacological inhibition of FABP7 reduces TM formation and increases sensitivity to TMZ, we propose that FABP7 inhibition, and possibly PKC inhibition, warrant further investigations as possible combinatorial approaches to improving clinical outcomes in GBM patients treated with TMZ. Using the updated drug-gene interaction database,^48^ 2 drugs were found to inhibit FABP7 (BPKDI and CRT 0066101), neither one of which currently has regulatory approval. Several drugs were found to interact with PKCα, including at least 2 inhibitory drugs with regulatory approval (quercetin, midostaurin). Bevacizumab, used for the treatment of recurrent GBM, also interacts with PKCα.

Finally, we found no consistent pattern between *MGMT* promoter methylation status (Supplementary Table 2), *MGMT* RNA levels in our patient-derived GBM neurosphere cultures (Supplementary Figure 8), and TMZ response. For example, A4-007 GBM neurospheres, derived from a patient tumor that was positive for *MGMT* promoter methylation, showed mid-range *MGMT* RNA levels and was resistant to TMZ treatment. Discrepancies in *MGMT* promoter methylation status, MGMT protein levels, and patient response to TMZ are common in GBM, likely reflecting GBM tumor heterogeneity.^49^ Neural stem-like cells which are selected for in GBM neurosphere cultures have already been shown to be particularly resistant to TMZ.^50^

In summary, we show that a subset of FABP7-expressing GBM neurosphere cultures produce TMs that serve as channels for rapid and directional migration of tumor cells and establishment of satellite GBM colonies. Our results indicate that FABP7 promotes TM formation through activation of PKC-mediated GAP43 phosphorylation. Importantly, we show that FABP7 inhibition improves survival in mice orthotopically injected with TM-forming GBM cells regardless of whether the mice are treated with radiation or not. We propose that FABP7-PKC-pGAP43 is a major signaling pathway in TM formation, and suggest that pharmacological inhibition of this pathway may be key to improved clinical outcomes for patients with GBM.

## Supplementary Material

noaf200_Supplementary_Tables_1-2_Figures_1-9

noaf200_Supplementary_Materials

## Data Availability

We provide links to all the databases accessed for the study.
