# Supplementary material for: Glioblastoma tumor microtubes and brain fatty acid-binding protein: Path to directional infiltration
Source: Neuro Oncol. 2025 Aug 28;27(12):3072–86. doi: 10.1093/neuonc/noaf200 (PMC12916726; doi:10.1093/neuonc/noaf200)
Supplement: noaf200_Supplementary_Tables_1-2_Figures_1-9 [file noaf200_supplementary_tables_1-2_figures_1-9.pdf]

Supplementary Methods

Transmission electron microscopy. A4-018 GBM cells in neurosphere medium were cultured on Aclar sheets until they formed robust TMs. The cells were fixed in 2% paraformaldehyde + 2.5% glutaraldehyde in 0.1M phosphate buffer overnight at 4ºC, and dehydrated in 30%, 50%, 70%, 90% and 100% ethanol solution as previously described[1]. Dehydration was followed by resin infiltration using Spurr's resin:100% ethanol at 1:4, 1:1 and 3:1 ratios. Cells were embedded in pure Spurr's resin followed by polymerization. Samples were cut into 100 nm sections and post-stained with 1% uranyl acetate for 15 minutes followed by 1% lead citrate for 4 minutes. TEM images were acquired on a JEOL 2100F TEM with Gatan Digital Microscope Suite Software (Digital Micrograph, Version 3.23).

Measurement of calcium flux. A4-007 GBM cells in neuroshere medium were plated on 35-mm glass bottom dishes (MatTek, Ashland, MA) and incubated at 37ºC in a humidified 5% CO_2 incubator for 4-5 days until microtube connection between neurospheres could be visualized. Fluo-4-AM (Cat. No. F14201; Thermo Fisher Scientific) at a final concentration of 1 μM was added to the cultures and incubated at room temperature for 30 minutes. Cells were then washed for 10 min and fresh neurosphere medium added. Laser-induced injury with a 405 nm laser set at 100% output was used to elicit transient Ca^{+2} flux. Fluo-4 AM emits a bright green signal upon binding to Ca^{+2}. The intracellular movement of Ca^{+2} within TM-connected neurospheres was monitored by live-cell fluorescence microscopy over a period of 250 seconds at 1

second intervals using a Zeiss LSM710 confocal microscope with a 40X/NA1.3 oil immersion objective.

Quantification of TMs from neurosphere cultures. Image analyses of TM formation upon FABP7 inhibition and PKC inhibition in A4-007 and A4-016 were performed in MATLAB (MathWorks). Image analyses were performed in MATLAB (MathWorks). First, the images were corrected for non-uniform background as previously detailed [2], using a wide Gaussian (standard deviation 10 pixels) to estimate the background image. Next, we used a segmentation strategy to establish a threshold value and the Sobel operator was applied to the image to get a binary gradient image mask of the neurospheres and their TMs[3,4]. Linear gaps in the gradient images were dilated using linear structuring elements, interior holes filled, and resultant boundary masks smoothened by eroding the image twice with a diamond structuring element. Segmentations were then manually inspected to ensure correct boundary detection. The number of protrusions and their lengths were extracted from these segmentations. Protrusiveness metric (pm) was computed using the area and perimeter of the segmentation boundary using the following formula: pm = 1 - ((A x 2)/(P x r)) where A=area, P=perimeter and r=radius of a circle.

Image analyses of TM formation upon shRNA-mediated PRKCA knockdown in A4-007 were performed using MetaXpress (Version 6.5, Molecular Devices, Sunnyvale, CA, USA). The following method was used for quantification of TMs in A4-007 control and PRKCA knockdown neurosphere cultures. Brightfield images were background corrected for uneven illuminations. Protrusions were then measured using the Neurite Outgrowth algorithm set at ≥15 μm to define significant processes. This algorithm detects

neurospheres and the protrusions attached to the neurospheres as processes. The segmentations of neurospheres versus protrusions were visually inspected for accuracy. The lengths and numbers of protrusions were extracted from the software module. Only the neurospheres with complete processes within the captured frame were analyzed.

Treatment of intracranially-injected NSG mice with SBFI-26 and radiation. TM-forming patient-derived A4-007 neurosphere cultures were orthotopically injected into the brains of NSG mice (100,000 cells/mouse) as described under Materials and Methods. Five weeks later, mice were randomly assigned to 4 groups (8 mice control no radiation; 9 mice FABP7 inhibitor SBFI-26 no radiation; 8 mice control with radiation; 8 mice FABP7 inhibitor SBFI-26 with radiation). Over the next 28 days, the 16 control mice were each intraperitoneally injected with 500 µL vehicle [50 µL DMSO + 450 µL Sulfobutyether-β-Cyclodextrin (SBE-β-CD) (Millipore Catalogue No. 1.42022; Sigma-Millipore) in 0.9% NaCl; 20% (w/v)] twice a week (9 injections in total). The 17 mice treated with SBFI-26 were each intraperitoneally injected with 15 mg/kg body weight SBFI-26 (total volume 500 µL for each injection) twice a week (9 injections in total). SBFI-26 (custom-prepared by MedChemExpress) was dissolved in DMSO at a concentration of 20 mM (8.45 mg/mL), aliquoted and stored at -80ºC. At the time of injection, SBFI-26 was slowly added to 20% SBE-β-CD while mixing at 37ºC until dissolved.

After vehicle or SBFI-26 treatment, mice from these two groups were randomly divided into 'no radiation' or 'radiation' groups. For radiation planning and calculation, we used MuriPlan (Xstrahl Inc. Sugar Hill, Georgia USA) and radiation was delivered using a Small Animal Radiation Research Platform (SARRP; Xstrahl). A treatment dose of 800

cGy was delivered in a single fraction of 220 kVp x-rays, using 5 X 5 mm^2 beams arranged in a dorso-ventral configuration. Beam angles were adjusted to minimize dose to the salivary glands. The experimental end-point was set at 135 days or when mice lost 20% of their body weight or showed signs of distress (changes in physical appearance, body condition, mobility, feeding, drinking and behavior). Mice were monitored twice a week until they reached 10% loss in body weight, at which time mice were monitored daily. One mouse in the control group was removed because it showed no sign of distress or weight loss after the experimental endpoint of 135 days was reached. Mouse survival comparisons among experimental groups with log rank test and Kaplan-Meier survival plot generation were carried out using Prism (Version 8.0.2).

1. Wang Y, Yasmin L, Li L, et al. DDX1 vesicles control calcium-dependent mitochondrial activity in mouse embryos. *Nat Commun.* 2022; 13(1):3794.

2. Githaka JM, Vega AR, Baird MA, Davidson MW, Jaqaman K, Touret N. Ligand-induced growth and compaction of CD36 nanoclusters enriched in Fyn induces Fyn signaling. *J Cell Sci.* 2016; 129(22):4175-4189.

3. Githaka JM, Tripathi N, Kirschenman R, et al. BAD regulates mammary gland morphogenesis by 4E-BP1-mediated control of localized translation in mouse and human models. *Nat Commun.* 2021; 12(1):2939.

4. Pandya V, Githaka JM, Patel N, et al. BIK drives an aggressive breast cancer phenotype through sublethal apoptosis and predicts poor prognosis of ER-positive breast cancer. *Cell Death Dis.* 2020; 11(6):448.

Supplementary Table 1: List of primers and shRNAs

Gene name		Sequence
**Semi-quantitative RT-PCR**		
FABP7	Forward (5’ to 3’)	TGGAGGCTTTCTGTGCTAC
	Reverse (5’ to 3’)	TAGGATAGCACTGAGACTTG
GAP43	Forward (5’ to 3’)	AAGCTCATAAGGCCGCAACC
	Reverse (5’ to 3’)	CTATGTTCTCTTCAGCTTGGC
TTYH1	Forward (5’ to 3’)	TTTCATCGCTGTCTACCTCATC
	Reverse (5’ to 3’)	CACCTGTACTCCTCCACAAAG
GAPDH	Forward (5’ to 3’)	ACCAGGGAGGGCTGCAGT
	Reverse (5’ to 3’)	CAGTTCGGAGCCCACACG
**Quantitative RT-PCR**		
FABP7	Forward (5’ to 3’)	AGAAACTGTAAGTCTGTTGTTAGCC
	Reverse (5’ to 3’)	TTACTTACCATAACCATTTTGCCAT
PRKCA	Forward (5’ to 3’)	TCCAGTGCCAAGTTTGCTGT
	Reverse (5’ to 3’)	CCACAGTGATCGCAGAAGGT
PRKCB	Forward (5’ to 3’)	GTGCATGAGGTCAAGAACCAC
	Reverse (5’ to 3’)	TCCCGAAGCCCCAGATGAA
GAP43	Forward (5’ to 3’)	ACCATGCTGTGCTGTATGAGA
	Reverse (5’ to 3’)	CTGAATTTTGGTTGCGGCCT
TTYH1	Forward (5’ to 3’)	TGGAGGAGTACAGGTGGCTG
	Reverse (5’ to 3’)	CAGCTCAGGACGAGAACCAG
GAPDH	Forward (5’ to 3’)	ACCAGGGAGGGCTGCAGT
	Reverse (5’ to 3’)	CAGTTCGGAGCCCACACG
18s rRNA	Forward (5’ to 3’)	AGGGCAGGGACTTAATCAACGC
	Reverse (5’ to 3’)	TCAGCGTGTGCCTACCCTAC
**shRNA target sequences**		
PRKCA #1		GACACAATTGTGCTCTATTTG
PRKCA #2		CTGAATATACTGGGCTATTTG
PRKCA #3		GCTGTACTTCGTCATGGAATA
PRKCA #4		CGAGGTGAAGGACCACAAATT

Supplementary Table 2. Clinical and limited genetic information on GBM tumors used in our study.

Patient	MGMT Methylation	IDH	Grade	ATRX	BRAF	PTEN	H3 Gene	TERT Promoter	TP53	Ip/19q	Comments
A4-004	Negative	WT	4	ND	ND	ND	ND	ND	ND	ND	
**A4-007***	**Positive**	**WT**	**4**	**RET**	**ND**	**ND**	**ND**	**ND**	**ND**	**ND**	**EGFR amplification**
A4-009	Negative	WT	4	RET	ND	ND	ND	ND	ND	ND	
A4-011	Negative	WT	4	RET	ND	ND	ND	ND	-	ND	EGFRvIII+
A4-012	Negative	WT	4	RET	ND	ND	ND	ND	-	ND	
**A4-016***	**Positive**	**WT**	**4**	**RET**	**-**	**-**	**-**	**C250T**	**-**	**ND**	
**A4-018***	**Positive**	**WT**	**4**	**RET**	**-**	**-**	**-**	**C228T**	**-**	**ND**	
A4-019	Negative	WT	4	RET	-	-	-	-	-	ND	EGFR, CDK4 and MDM2 amplification

MGMT promoter methylation status was identified using methylation-specific PCR. RET indicates retention of nuclear ATXR. "-" means that no mutations were identified in *BRAF*, *PTEN*, histone 3 (H3), *TERT* promoter, or *TP53*. TERT promoter analysis identified C → T mutations at C228 and C250 in three samples. ND indicates Not Done. *EGFR* amplification was noted in A4-007 and A4-019.

*Experiments designed to study TM formation *in vitro* were carried out with A4-007, A4-016 and A4-018 (bold).

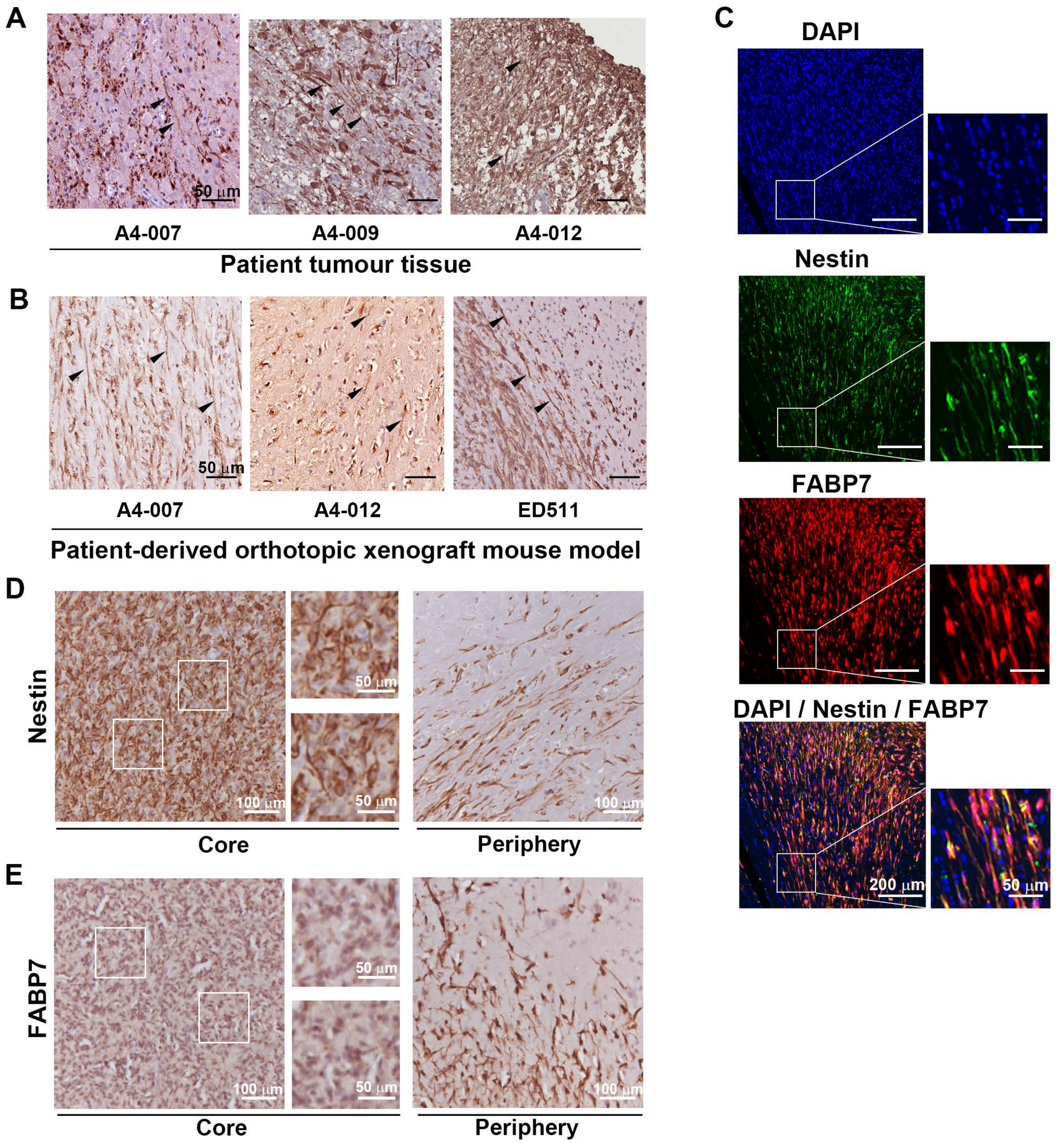

Supplementary Figure 1. FABP7 is expressed in long extended GBM protrusions. (A-B) Immunohistochemical analysis of paraffin-embedded GBM tumor tissue sections from three patients (A4-007, A4-009, A4-012) (A) and paraffin-embedded GBM tissue sections from the brains of NSG mice orthotopically injected with human GBMs A4-007, A4-012 and ED511 (B) immunostained with anti-FABP7 antibody. Slides were counterstained with hematoxylin. Black arrowheads point to sites of FABP7 expression in TMs. Scale bars = 50 µm. (C) Representative images showing co-immunostaining of nestin and FABP7 in A4-007 mouse xenograft tumor tissues. Area in square is magnified on the right. Scale bars 200 µm or 50 µm as indicated. (D,E) Immunohistochemical analysis of A4-007 orthotopic mouse xenograft tumor tissues immunostained with anti-nestin antibody (D) or anti-FABP7 antibody (E). Representative images are shown of the tumor core and tumor periphery (infiltrative zone). Areas in squares are magnified on the right. Scale bars: 100 µm or 50 µm as indicated.

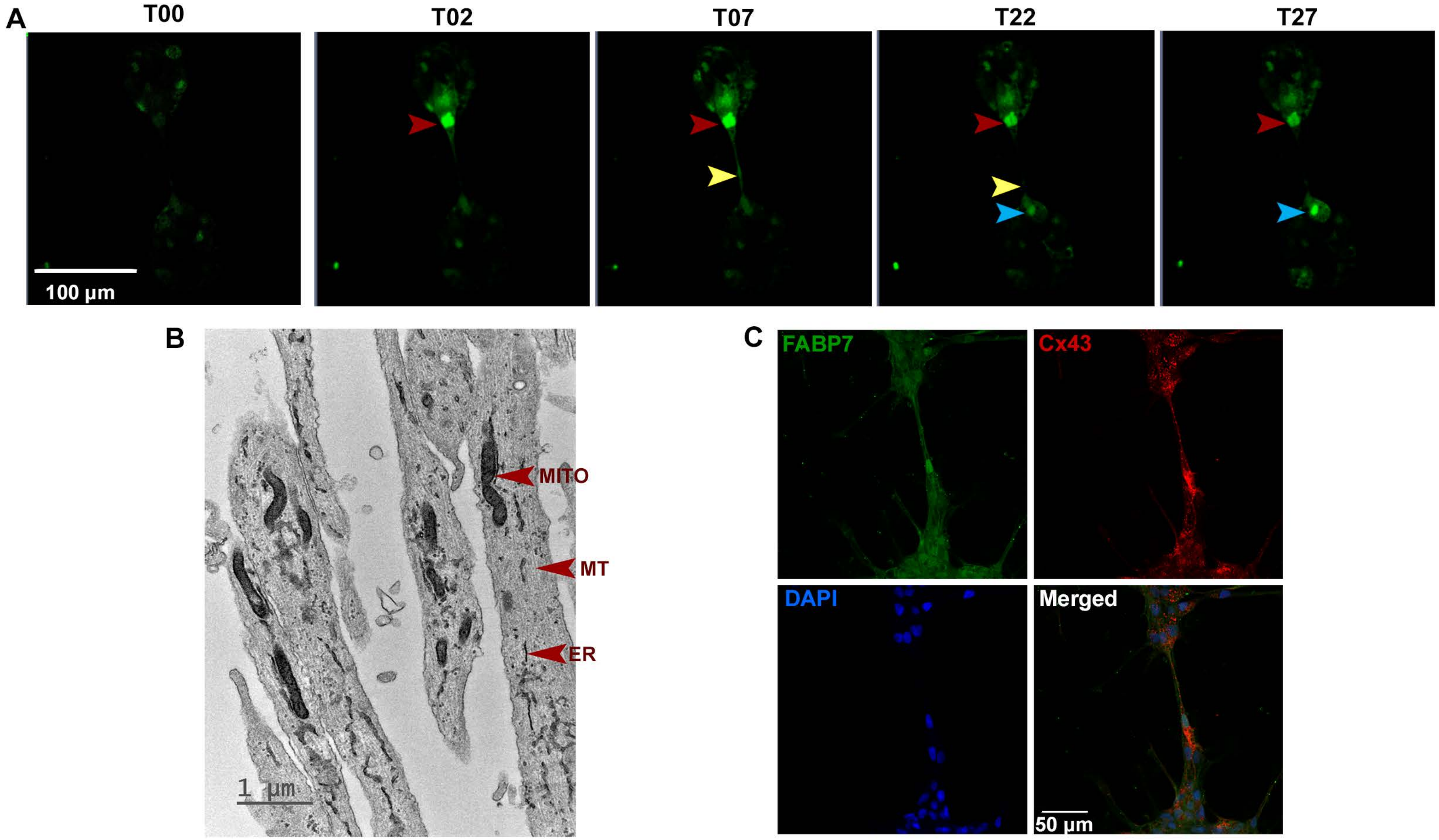

Supplementary Figure 2. Characterization of TMs in GBM neurosphere cultures. (A) Montage of five time-lapse photos taken at T00, T02, T07, T22 and T27 (seconds) (start of Video 1) showing calcium flux between two neurospheres (red and blue arrows) linked by a TM (yellow arrow) in culture of A4-018 GBM neurospheres. (B) Transmission electron micrograph of TMs produced by a culture of A4-018 neurospheres. MITO: mitochondria; MT: microtubules: ER: endoplasmic reticulum. (C) Images showing co-immunostaining of FABP7 and Cx43 across a TM linking two neurospheres in an A4-007 GBM neurosphere culture.

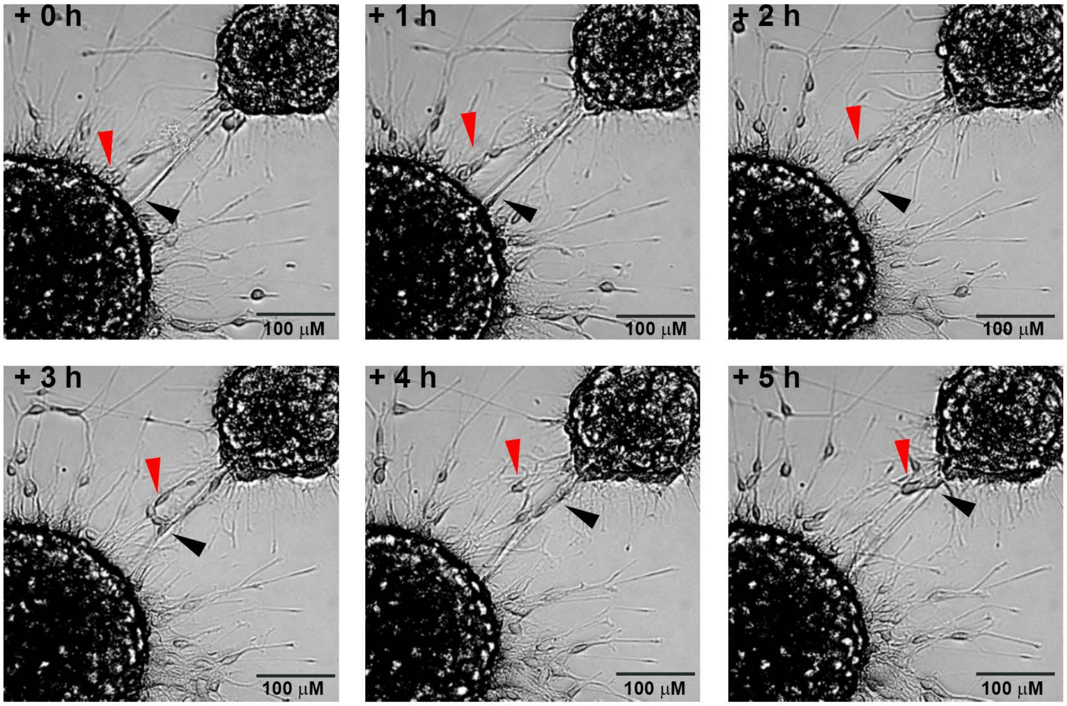

Supplementary Figure 3. Cell movement along tumor microtubes in A4-016 GBM neurospheres. Representative bright field timelapse images showing A4-016 cells migrating along TMs.

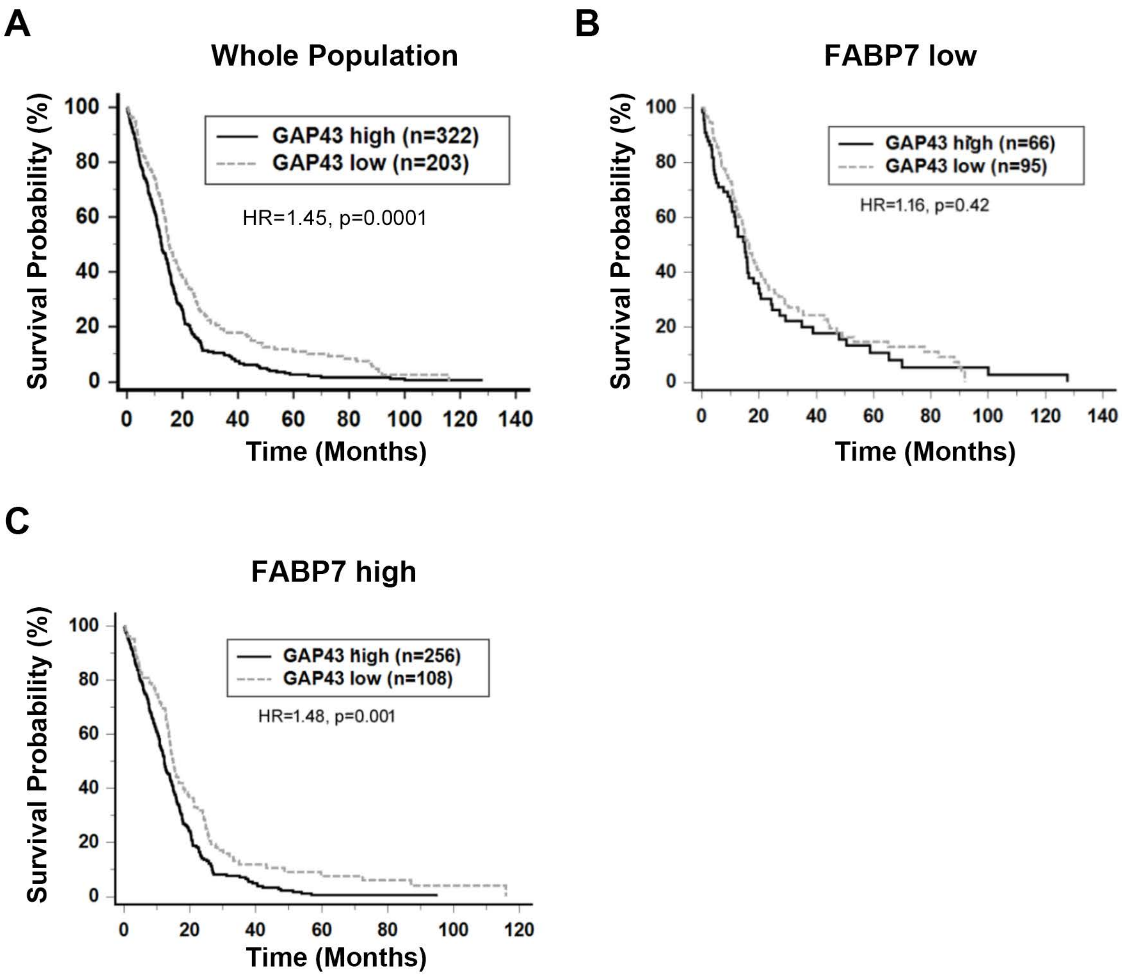

Supplementary Figure 4. Prognostic significance of GAP43 and FABP7 in GBM patients. Patient overall survival curves comparing GAP43-high vs GAP43-low patients in a GBM patient cohort (HG-U133A dataset) in whole population (A), FABP7-low population (B), and FABP7-high population (C). The median value of FABP7 expression level was used as a cutoff.

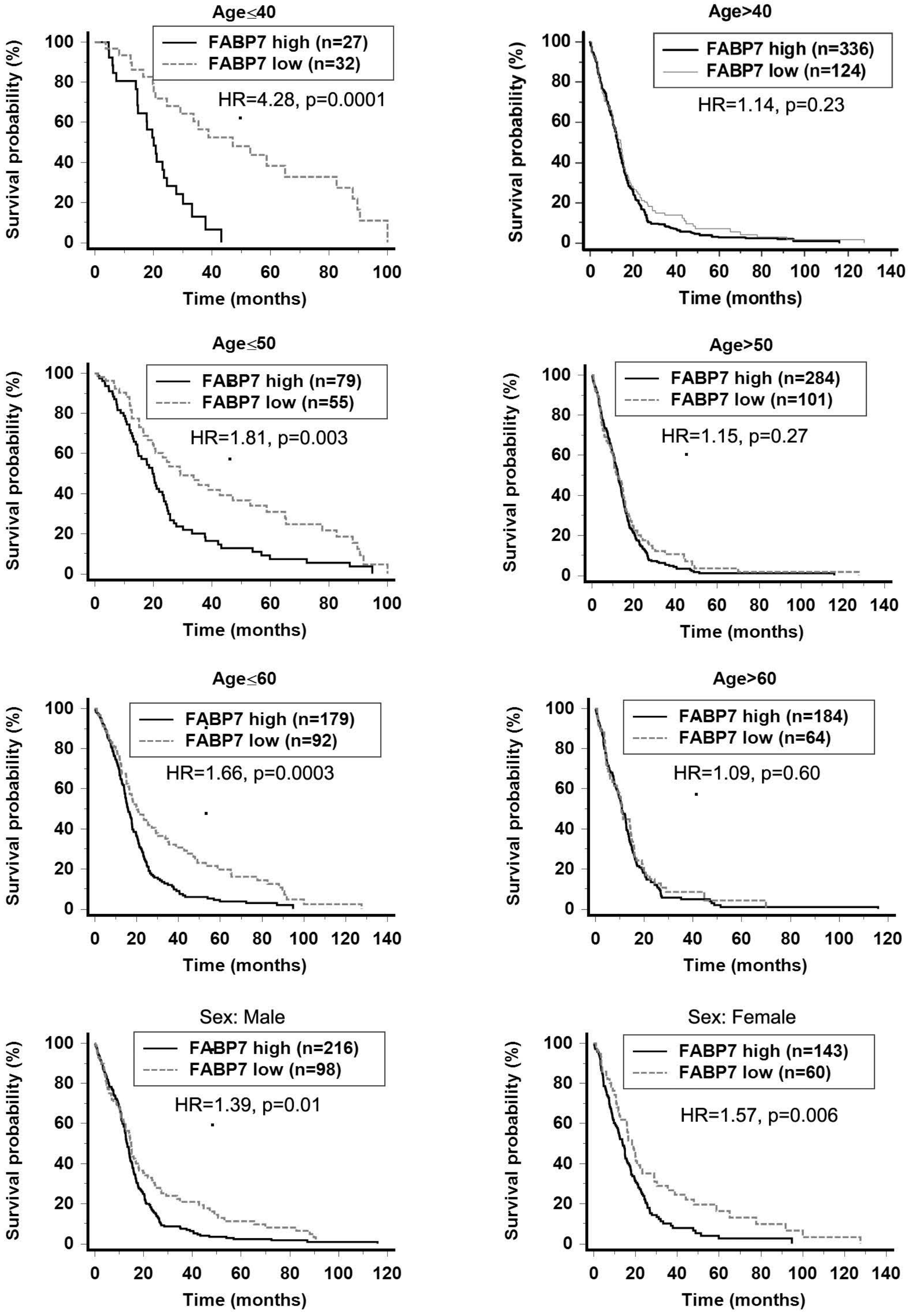

Supplementary Figure 5. Prognostic significance of FABP7 in a GBM patient cohort as a function of patient age and sex. Gene expression and clinical record data of a human GBM dataset (HG-U133A, TCGA-GBM) was retrieved from GlioVis (http://gliovis.bioinfo.cnio.es). Log-rank patient survival curves were generated with the Kaplan-Meier survival curve procedure in MedCalc (Version 23.0.2). HR denotes hazard ratio.

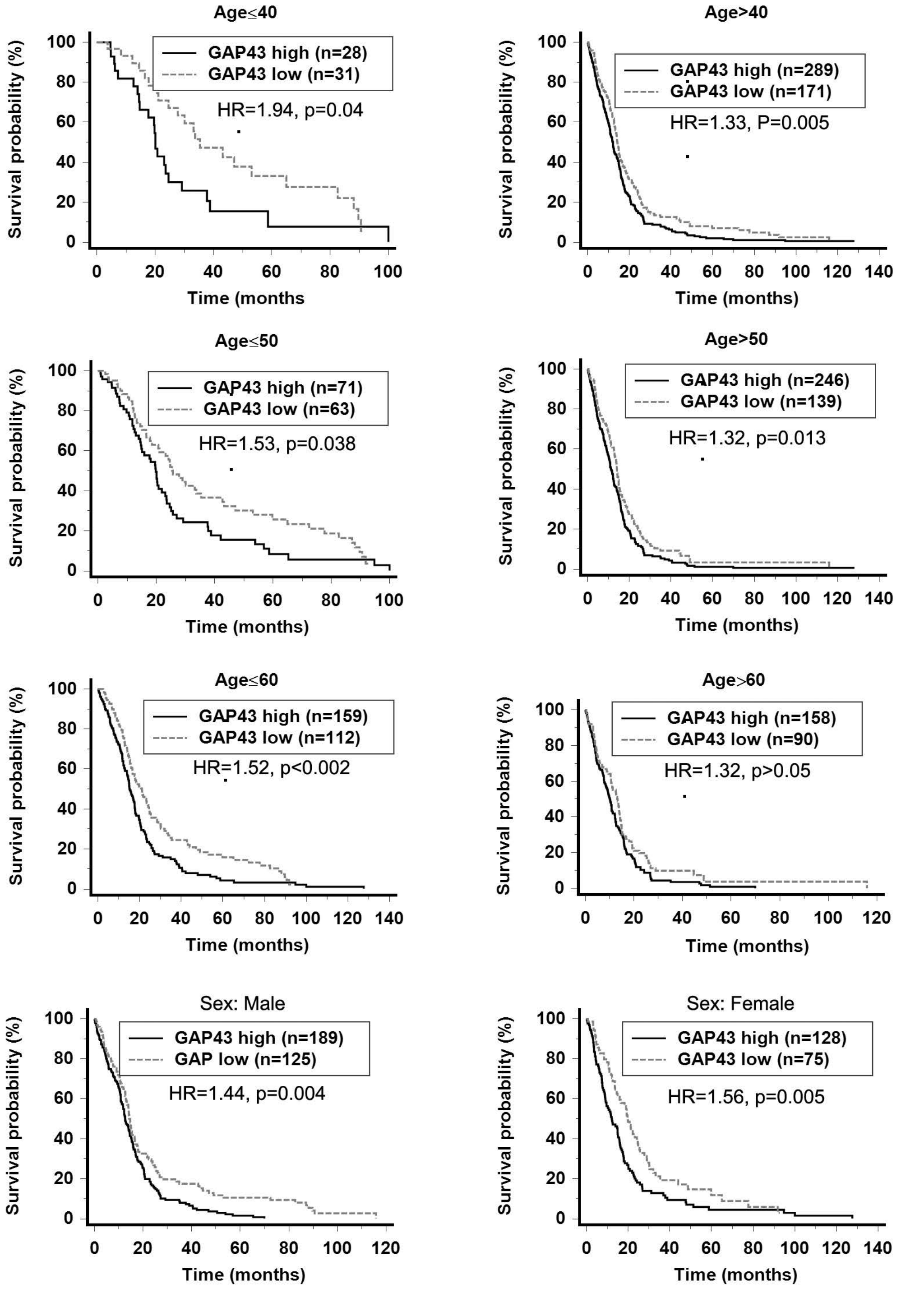

Supplementary Figure 6. Prognostic significance of GAP43 in a GBM patient cohort as a function of patient age and sex. Gene expression and clinical record data of a human GBM dataset (HG-U133A, TCGA-GBM) was retrieved from GlioVis (http://gliovis.bioinfo.cnio.es). Log-rank patient survival curves were generated with the Kaplan-Meier survival curve procedure in MedCalc (Version 23.0.2). HR denotes hazard ratio.

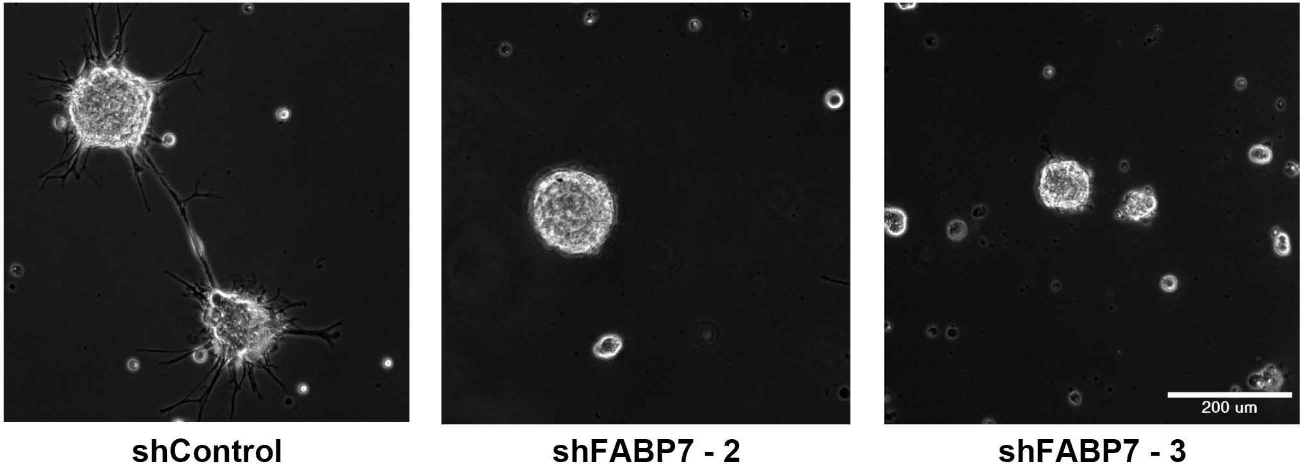

Supplementary Figure 7. FABP7 knockdown inhibits tumor microtube formation. Representative bright field images showing that FABP7 depletion in A4-007 GBM neurosphere cultures inhibits TM formation.

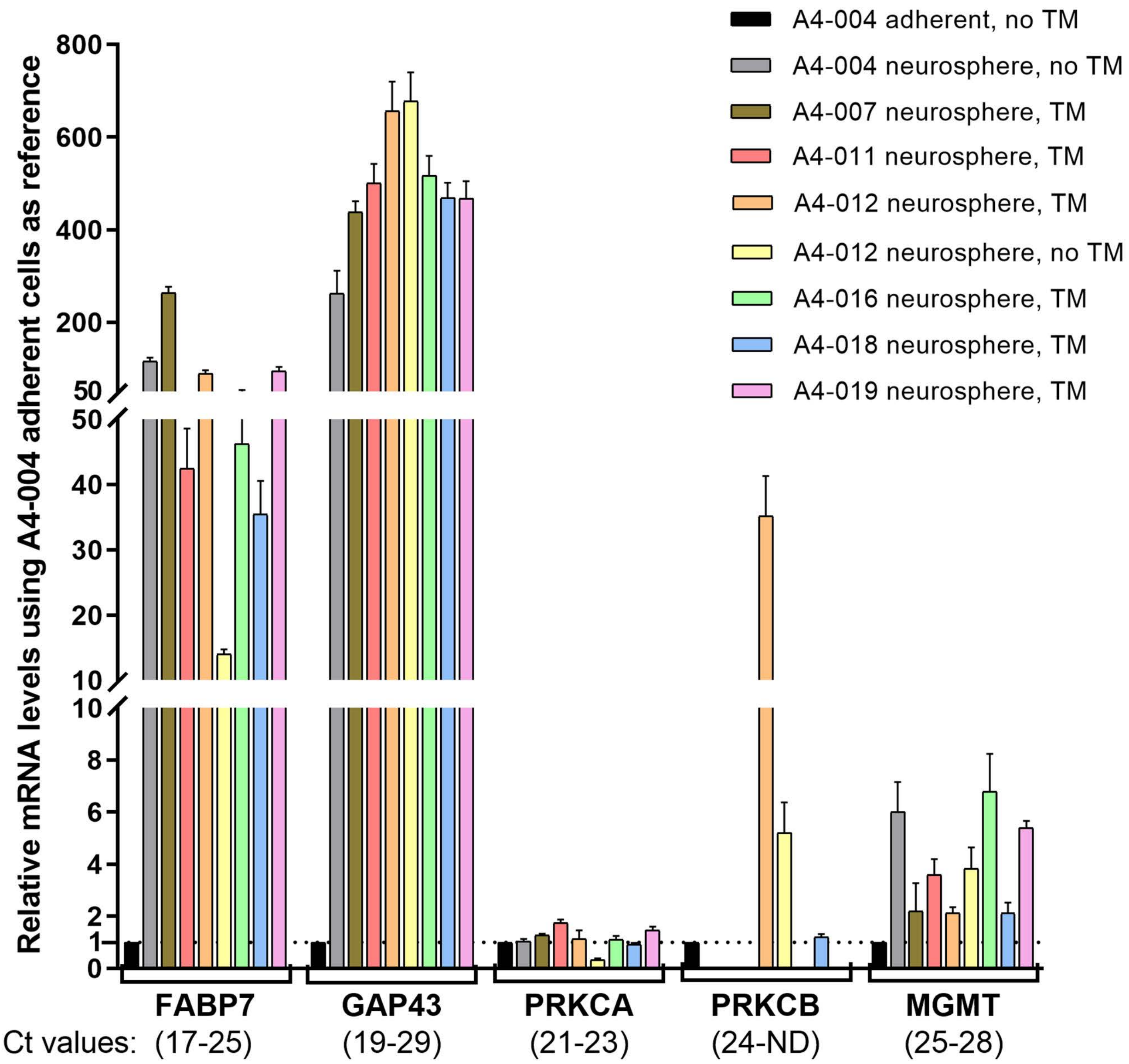

Supplementary Figure 8: Gene expression analysis of GBM cultures. Total RNA was isolated from 6 GBM neurosphere cultures that form TMs *in vitro* (A4-007, A4-011, A4-012, A4-016, A4-018, A4-019 neurosphere, TM), 1 GBM neurosphere culture unable to form TMs (A4-004 neurosphere), 1 GBM neurosphere culture initially able to form MTs, but which lost the ability to form MTs after several months in culture (A4-012 neurosphere, no TM), and 1 GBM cell line cultured in DMEM medium containing 10% fetal calf serum (A4-004 adherent, no TM). cDNA was synthesized using 5 µg RNA, random primers and Superscript reverse transcriptase II. RT-qPCR was carried out using 1:100 of the cDNA and primers for *FABP7*, *GAP43*, *PRKCA*, *PRKCB* and *MGMT*. mRNA levels are relative to 18s rRNA. The range of Ct values, indicated for each set of primers, is an indication of RNA abundance with lower Ct values indicating increased abundance. Error bars represent standard deviation of three sets of measurements.

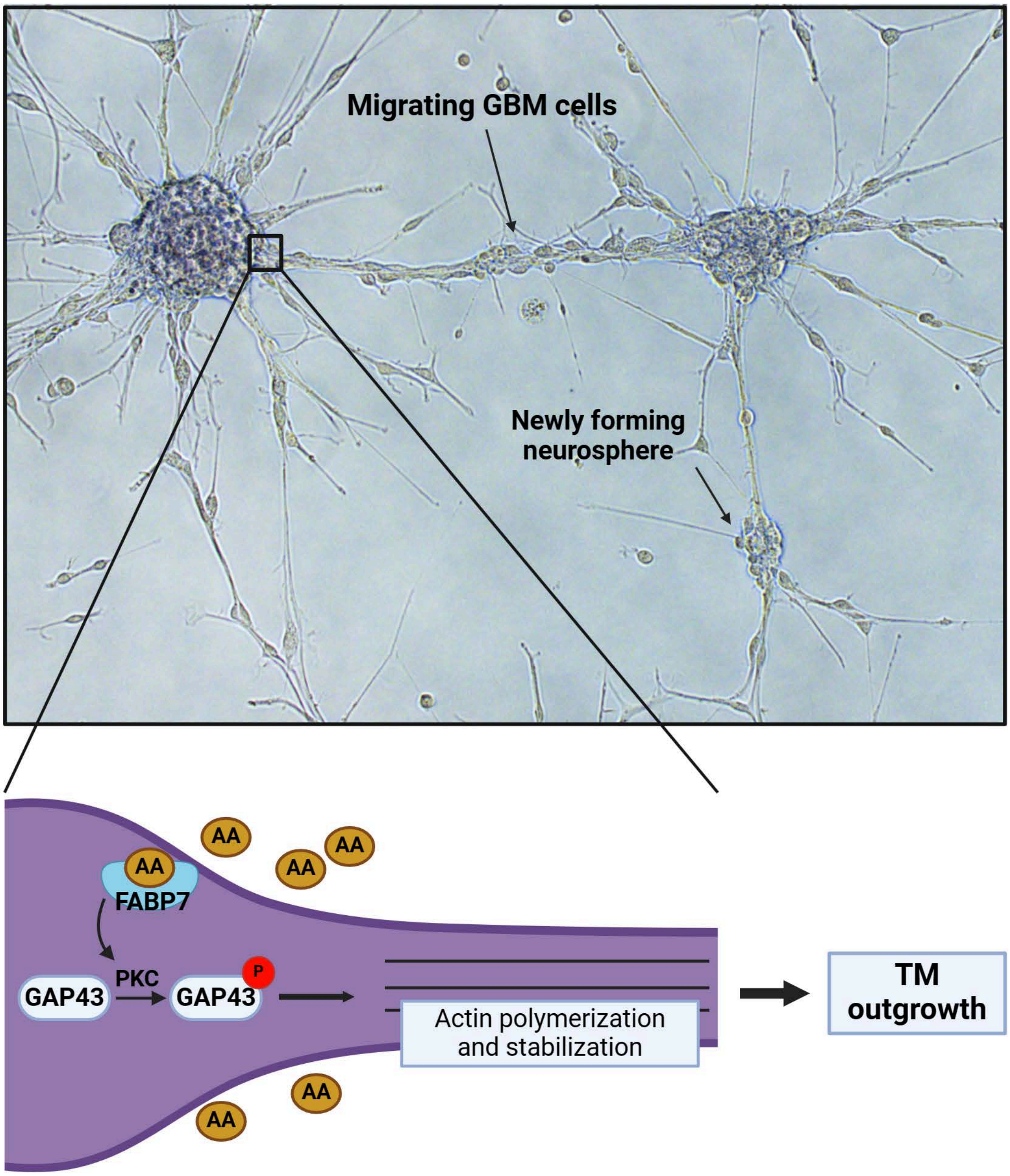

Supplementary Figure 9. Proposed role for FABP7 in tumor microtube formation. (Top panel) Photomicrograph of cultured GBM neurospheres connected to each other via TMs, with TMs providing structural guidance for GBM cell migration. (Bottom panel) Schematic representation of PKC activation through arachidonic acid (AA)-bound FABP7. Activated PKC phosphorylates GAP43 which promotes TM outgrowth.
